# Association of Age With Risk of Kidney Failure in Adults With Stage IV Chronic Kidney Disease in Canada

**DOI:** 10.1001/jamanetworkopen.2020.17150

**Published:** 2020-09-18

**Authors:** Pietro Ravani, Rob Quinn, Marta Fiocco, Ping Liu, Huda Al-Wahsh, Ngan Lam, Brenda R. Hemmelgarn, Braden J. Manns, Matthew T. James, Yves Joanette, Marcello Tonelli

**Affiliations:** 1 Department of Medicine, Cumming School of Medicine, University of Calgary, Calgary, Alberta, Canada; 2 Mathematical Institute, Medical Statistics Section, Department of Biomedical Data Science, Leiden University, Leiden University Medical Center, Leiden, the Netherlands; 3 Faculty of Medicine, Université de Montréal and Centre de Recherche de l’Insitut Universitaire de Montréal, Montréal, Québec, Canada

## Abstract

**Importance:**

With population aging, the burden of many age-related chronic conditions, including kidney failure, is increasing globally.

**Objective:**

To investigate the risks of kidney failure and death in adults with incident stage IV chronic kidney disease (CKD).

**Design, Setting, and Participants:**

This population-based cohort study obtained data recorded between July 30, 2002, and March 31, 2014, from the linked laboratory and administrative data set of Alberta Health in Alberta, Canada. All adults of the province of Alberta with stage IV CKD (estimated glomerular filtration rate [eGFR] of 15-30 mL/min/1.73 m^2^) were eligible for inclusion. Included individuals were followed up from study entry until the date of kidney failure, death, or censoring, whichever occurred first. Observations were censored at the date of emigration from the province, the study end date (March 31, 2017), or at 10 years after study entry. Data analyses were performed from January 2020 to June 2020.

**Main Outcomes and Measures:**

The primary outcome was kidney failure, defined as the earlier of either renal replacement (dialysis or kidney transplant) initiation or severe kidney impairment (eGFR <10 mL/min/1.73 m^2^). Incidence of stage IV CKD in Alberta was examined over time, along with the association between age at study entry and the competing risks of kidney failure and death. Cumulative incidence functions (95% CIs) were estimated to summarize absolute risks over time across categories of age, accounting for sex, diabetes, cardiovascular disease, eGFR, and albuminuria.

**Results:**

The study included 30 801 adults (mean [SD] age, 76.8 [13.3] years; 17 294 women [56.1%]) with stage IV CKD. Of these, 5511 developed kidney failure (17.9%) and 16 285 died (52.9%). The incidence rate of stage IV CKD increased sharply with advancing age; the absolute risk of kidney failure decreased with advancing age, and the risk of death increased, especially in those aged 85 years or older. Compared with the 5-year risk of death, the 5-year risk of kidney failure was higher in people younger than 65 years, similar in people aged 65 to 74 years, and lower for older age groups. For those aged 75 years or older, the risk of death was much higher than the risk of kidney failure: 6-fold higher among those aged 75 to 84 years (0.51 [95% CI, 0.5-0.52] vs 0.09 [95% CI, 0.08-0.09]) and 25-fold higher among those aged 85 years or older (0.75 [95% CI, 0.74-0.76] vs 0.03 [95% CI, 0.02-0.03]). The risk of death was higher than the risk of kidney failure by 24-fold among those aged 85 to 94 years (0.73 [95% CI, 0.72-0.74] vs 0.03 [95% CI, 0.02-0.03]) and by 149-fold among those aged 95 years or older (0.89 [95% CI, 0.87-0.92] vs <0.01 [95% CI, <0.01 to 0.01]).

**Conclusions and Relevance:**

This study found that, although the incidence rate of stage IV CKD increased with advancing age, the absolute risk of kidney failure decreased. Unlike other age-related conditions, the expected increase in the burden of kidney failure in the older adults may be less dramatic than expected.

## Key Points

**Question:**

Does the risk of kidney failure vary with age in adults with severe chronic kidney disease (CKD)?

**Findings:**

In this population-based cohort study of approximately 4 million people, the incidence of severe CKD increased sharply with older age. In 30 801 adults who developed stage IV CKD, on average, death was 3 times more likely to occur than kidney failure, 6 times more likely among those aged 75 to 84 years, and 25 times more likely among those aged 85 years or older.

**Meaning:**

The findings of this study suggest that the increasing burden of severe CKD that accompanies population aging may not translate into increased demand for treatment of kidney failure in older adults.

## Teaser

This cohort study examines the competing risks of death and kidney failure in an aging population with or without age-related comorbid conditions.

## Introduction

Because of lower birth rates and longer life expectancy, the proportion of older adults in the general population has been steadily increasing.^1^ By 1 estimate, the number of people older than 80 years will triple between 2019 and 2050, from 143 million to 425 million, worldwide.^2^ Chronic kidney disease (CKD), which is defined as an estimated glomerular filtration rate (eGFR) of less than 60 mL/min/1.73 m^2^, affects 10% to 16% of the general population worldwide^3^ and is associated with death and kidney failure.^4^ The prevalence of CKD increases with age (from 4% at younger than 40 years to 47% at 70 years or older^5^), as do more severe CKD stages characterized by lower eGFR and worse outcomes.^6^ As in other populations, among people with CKD, the prevalence of comorbidity and the risk of adverse health outcomes increase in parallel with age.^7,8^

With population growth and aging, the number of people with chronic medical conditions is expected to rise worldwide.^9^ For example, the global number of people living with dementia more than doubled from 1990 to 2016,^10^ and approximately 60% of people aged 80 years or older have at least 3 chronic disorders.^11^ However, death is a competing risk for developing kidney failure,^12^ and some but not all older people with advanced CKD will live long enough to develop kidney failure. Studies that assessed the association between age and kidney failure in people with CKD reported rates or relative hazards^13,14,15^ from which absolute risks cannot be obtained directly in the presence of competing events.^12^ In addition, with few exceptions,^13,14^ previous studies were largely based on selected cohorts of people receiving nephrology care,^15^ who may differ in clinically important ways from those not receiving nephrology care.^16^ Given that treatments for kidney failure, including renal replacement with dialysis or kidney transplant, are resource intensive, data on the association of population aging with the burden of kidney failure are potentially valuable to policymakers.

Although the prevalence of a disease in a population depends on both disease duration and new cases, high incidence rates require immediate attention because they have rapid implications for health systems and are potentially suitable for prevention. We conducted a population-based study to investigate the risks of kidney failure (sustained eGFR <10 mL/min/1.73 m^2^ or initiation of renal replacement) and death in adults with severe CKD (stage IV CKD, defined as eGFR of 15-30 mL/min/1.73 m^2^), a major risk factor of and precursor to kidney failure. We assessed how these 2 competing risks vary by a person’s age, focusing especially on older people with or without comorbid conditions that are often associated with CKD (diabetes and cardiovascular disease [CVD]).

## Methods

This population-based cohort study used linked provincial administrative and laboratory data from the Alberta Health database (eMethods in the Supplement). The institutional review boards at the University of Alberta and University of Calgary approved this study with a waiver of participant consent, because the study used deidentified data. We followed the Reporting of Studies Conducted Using Observational Routinely-Collected Health Data (RECORD) statement.^17^

### Population

All Alberta, Canada, residents aged 18 years or older with stage IV CKD (eGFR of 15-30 mL/min/1.73 m^2^) were eligible for inclusion. We calculated eGFR using the Chronic Kidney Disease Epidemiology Collaboration equation, with serum creatinine values standardized to isotope dilution mass spectrometry traceable methods.^18^ Data on race/ethnicity are not routinely collected in Alberta. Given the low prevalence of people in the province who self-identified as being Black individuals (<3%), we calculated eGFR with the assumption that all adults were White individuals, as was done in our previous work^6,12,13^ (eMethods in the Supplement). To minimize the inclusion of people with acute kidney injury, unstable clinical conditions, or prevalent cases of stage IV CKD and to maximize the inclusion of incident cases, we applied a moving-average eGFR method to identify stage IV CKD using outpatient laboratory measures recorded between July 30, 2002, and March 31, 2014 (eMethods in the Supplement).^19^ We used the date of the last eGFR measurement included in the calculation of the mean (index eGFR) to establish study entry (index date). We excluded those who had received any renal replacement before study entry or had 1 or more outpatient eGFR of less than 15 mL/min/1.73 m^2^ before the episode qualifying for study entry.

### Independent Variables, Exposure, and Outcomes

We considered the following clinical characteristics associated with death or kidney failure: lower index eGFR, more severe albuminuria, diabetes, and CVD. Cardiovascular disease was described as 1 or more of the following conditions: congestive heart failure, myocardial infarction, stroke or transient ischemic attack, or peripheral vascular disease (amputation or peripheral revascularization). We used validated coding algorithms applied to physician claims and hospitalization data to specify comorbidities (eMethods in the Supplement).^20^ We used the most recent albuminuria values (on or within the 2 years preceding the index date), with the following types of measurement in descending order of preference: albumin to creatinine ratio, protein to creatinine ratio, and urine dipstick. We categorized albuminuria as normal, moderate, severe, or unmeasured.^21^

We assessed the association between age and kidney failure and death. The primary outcome was kidney failure, which was defined as the earlier of either renal replacement (dialysis or kidney transplant) initiation or severe kidney impairment (eGFR <10 mL/min/1.73 m^2^). We identified renal replacement on the basis of at least 1 inpatient or outpatient physician claim (eTable 1 in the Supplement). For the eGFR criterion, we applied the moving-average method^19^ (eMethods in the Supplement).

We followed individuals from study entry until the date of kidney failure, death, or censoring, whichever occurred first. We censored observations at the date of emigration from the province, the study end date (March 31, 2017), or at 10 years after study entry. To minimize bias in outcome ascertainment, we censored observations at 1.5 years from an eGFR measurement, if no subsequent measurement was available within 1.5 years of this eGFR.

### Statistical Analysis

We estimated the incidence of stage IV CKD and renal replacement using data from Statistics Canada.^22^

To study the risk of kidney failure, accounting for the competing risk of death and the risk of death without kidney failure, we estimated the unadjusted (Aalen-Johansen method) and adjusted (model-based) cumulative incidence functions and their 95% CIs. We used semiparametric regression to model cause-specific hazards and to estimate model-based cumulative incidence functions.^23^ We summarized data graphically, by age category in unadjusted or stratified analyses (<65, 65-74, 75-84, and ≥85 years), and subdivided the oldest group into 85 to 94 years and 95 years or older. In adjusted analyses, we treated age as a continuous variable and summarized data at age values of 60 to 95 years, in 5-year steps. In all analyses, we estimated risks across 4 mutually exclusive categories: (1) people without diabetes and CVD, (2) people with diabetes and without CVD, (3) people without diabetes and with CVD, and (4) people with diabetes and CVD. In model-based risk estimates, we considered the most likely value of albuminuria and eGFR in the sample and presented data by sex.

We tested the consistency of the findings in several sensitivity analyses (eMethods in the Supplement). R, version 4 (R Foundation for Statistical Computing), was used for all analyses. Data analyses were performed from January 2020 to June 2020.

## Results

A total of 30 801 individuals with stage IV CKD were included in the study (eFigure 1 in the Supplement), of whom 17 294 were women (56.1%) and 13 507 were men (43.9%) with a mean (SD) age of 76.8 (13.3) years (Table). Mean (SD) eGFR varied from 25.6 (3.8) mL/min/1.73 m^2^ in people younger than 65 years to 26.2 (3.3) mL/min in those 85 years or older. The older age categories were composed of fewer men (≥85 years: 2906 [32.6%] vs <65 years: 2794 [54.7%]), fewer people with diabetes (≥85 years: 2738 [30.7%] vs <65 years: 2758 [54.7%]) or severe albuminuria (≥85 years: 964 [10.8%] vs <65 years: 2718 [53.2%]), and more people with cardiovascular disease (≥85 years: 6188 [69.4%] vs <65 years: 1807 [35.4%]) or people who were missing information on albuminuria (≥85 years: 1331 [14.9%] vs <65 years: 270 [5.3%]).

**Table.** Clinical Characteristics at Baseline

Characteristic	No. (%)				
	All	Age group			
		<65 y	65-74 y	75-84 y	≥85 y
No.	30 801	5107	5792	10 985	8917
Age, mean (SD), y	76.8 (13.3)^a^	53.4 (10.2)	70.6 (2.9)	80.3 (2.8)	89.9 (3.7)
Men	13 507 (43.9)	2794 (54.7)	2942 (50.8)	4865 (44.3)	2906 (32.6)
eGFR, mean (SD), mL/min/1.73 m^2^	26.2 (3.4)	25.6 (3.8)	26.3 (3.4)	26.5 (3.3)	26.2 (3.3)
Albuminuria, missing	2978 (9.7)	270 (5.3)	382 (6.6)	995 (9.1)	1331 (14.9)
A1^b^	13 318 (43.2)	1093 (21.4)	2190 (37.8)	5278 (48)	4757 (53.3)
A2^b^	7091 (23)	1026 (20.1)	1464 (25.3)	2736 (24.9)	1865 (20.9)
A3^b^	7414 (24.1)	2718 (53.2)	1756 (30.3)	1976 (18)	964 (10.8)
Diabetes	13 906 (45.1)	2758 (54)	3403 (58.8)	5007 (45.6)	2738 (30.7)
CVD	18 063 (58.6)	1807 (35.4)	3188 (55)	6880 (62.6)	6188 (69.4)
MI	3692 (12)	360 (7)	684 (11.8)	1514 (13.8)	1134 (12.7)
CHF	13 073 (42.4)	1132 (22.2)	2211 (38.2)	4957 (45.1)	4773 (53.5)
Stroke or TIA	7865 (25.5)	716 (14)	1301 (22.5)	2999 (27.3)	2849 (32)
Peripheral vascular disease	2285 (7.4)	258 (5.1)	535 (9.2)	920 (8.4)	572 (6.4)

Abbreviations: CHF, congestive heart failure; CVD, cardiovascular disease; eGFR, estimated glomerular filtration rate; MI, myocardial infarction; TIA, transient ischemic attack.

^a^ Median (interquartile range) age was 80 (70-86) years.

^b^ A1, A2, and A3 are categories of proteinuria, which were calculated according to the following KDIGO (Kidney Disease Improving Global Outcomes) recommendations: A1 = albuminuria less than 30 mg/24 hours, or proteinuria less than 150 mg/24 hours, or albumin-creatinine ratio (ACR) less than 3 mg/mmol (<30 mg/g), or protein-creatinine ratio (PCR) less than 15 mg/mmol (<150 mg/g), or protein reagent strip negative to trace value. A2 = albuminuria 30 to 300 mg/24 hours, or proteinuria 150 to 500 mg/24 hours, or ACR 3 to 30 mg/mmol (30-300 mg/g), or PCR 15 to 50 mg/mmol (150-500 mg/g), or protein reagent strip trace to positive value. A3 = albuminuria greater than 300 mg/24 hours, or proteinuria greater than 500 mg/24 hours, or ACR greater than 30 mg/mmol (>300 mg/g), or PCR greater than 50 mg/mmol (>500 mg/g), or protein reagent strip positive value or greater.

**Figure 1.**
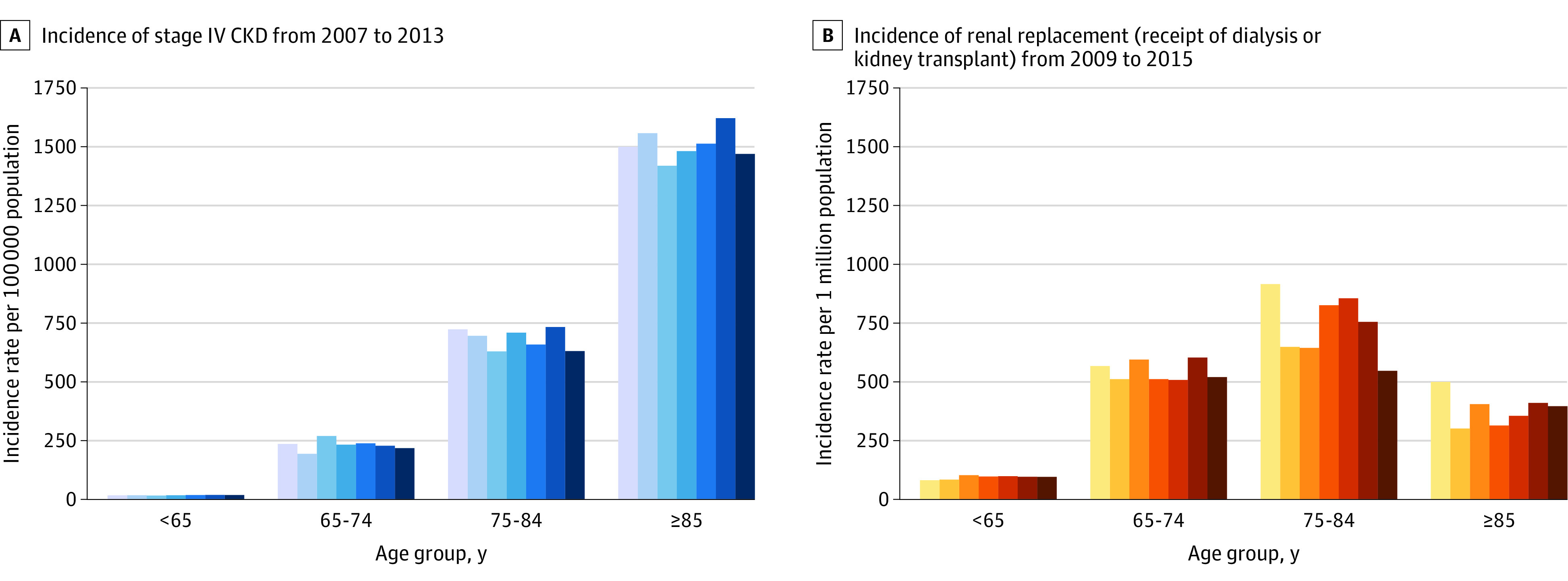
Yearly Incidence Rate of Stage IV Chronic Kidney Disease (CKD) and Renal Replacement in Alberta, Canada Each color band represents 1 year. Shading of the color bands indicates successive years, from the first year (lightest) to the last year (darkest).

Yearly incidence of stage IV CKD did not vary during the study period but increased sharply with older age (Figure 1A). Approximately 20 new cases per 100 000 person-years were found among those younger than 65 years, 250 cases among those aged 65 to 74 years, 750 cases among those aged 75 years to 84 years, and more than 1500 cases among those aged 85 years or older. The incidence of kidney failure treated with renal replacement increased with age until 84 years (from 100 per million population at age <65 years to 750 per million population at age 75-84 years) and decreased in people aged 85 years or older (250 per million population) (Figure 1B).

### Crude Risks

**Figure 2.**
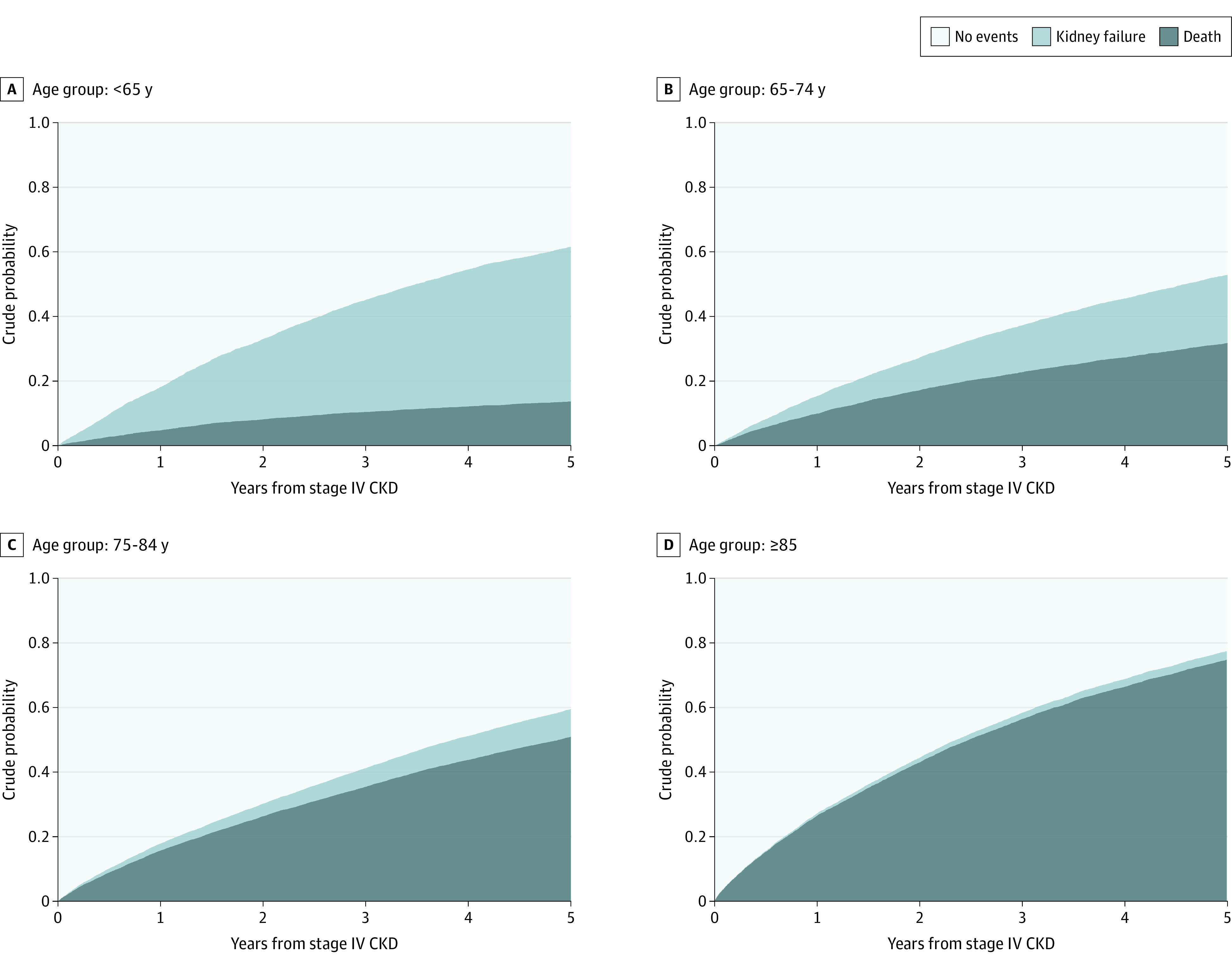
Observed Probabilities Over Time Stacked cumulative incidence functions by age in years are shown. The number-at-risk data are included in eFigure 2 in the Supplement. CKD indicates chronic kidney disease.

During follow-up (n = 106 447 person-years at risk), 5511 individuals (17.9%, or 5.2 per 100 person-years) developed kidney failure (4758 received renal replacement) and 16 285 people (52.9%, or 15.3 per 100 person-years) died without kidney failure (eFigure 2 in the Supplement). With advancing age, the risk of kidney failure decreased, and the risk of death increased. The risk of kidney failure was greater than the risk of death in people younger than 65 years but was lower than the risk of death for all other age groups (Figure 2). The 2 risks (95% CI) were closest in the 65 to 74 years age category. For those aged 75 years or older, the risk of death was much higher than the risk of kidney failure: 6-fold higher among those aged 75 to 84 years (0.51 [95% CI, 0.5-0.52] vs 0.09 [95% CI, 0.08-0.09]) and 25-fold higher among those aged 85 years or older (0.75 [95% CI, 0.74-0.76] vs 0.03 [95% CI, 0.02-0.03]).

When further subdivided into those aged 85 to 94 years and those aged 95 years or older, the risk of death was higher than the risk of kidney failure by 24-fold among those aged 85 to 94 years (0.73 [95% CI, 0.72-0.74] vs 0.03 [95% CI, 0.02-0.03]) and by 149-fold among those aged 95 years or older (0.89 [95% CI, 0.87-0.92] vs <0.01 [95% CI, <0.01 to 0.01]). The risk of kidney failure without renal replacement was low in all age groups but accounted for an increasing proportion of the total risk of kidney failure among those in older age categories (eFigures 3 and 4 in the Supplement).

### Stratified Risks

**Figure 3.**
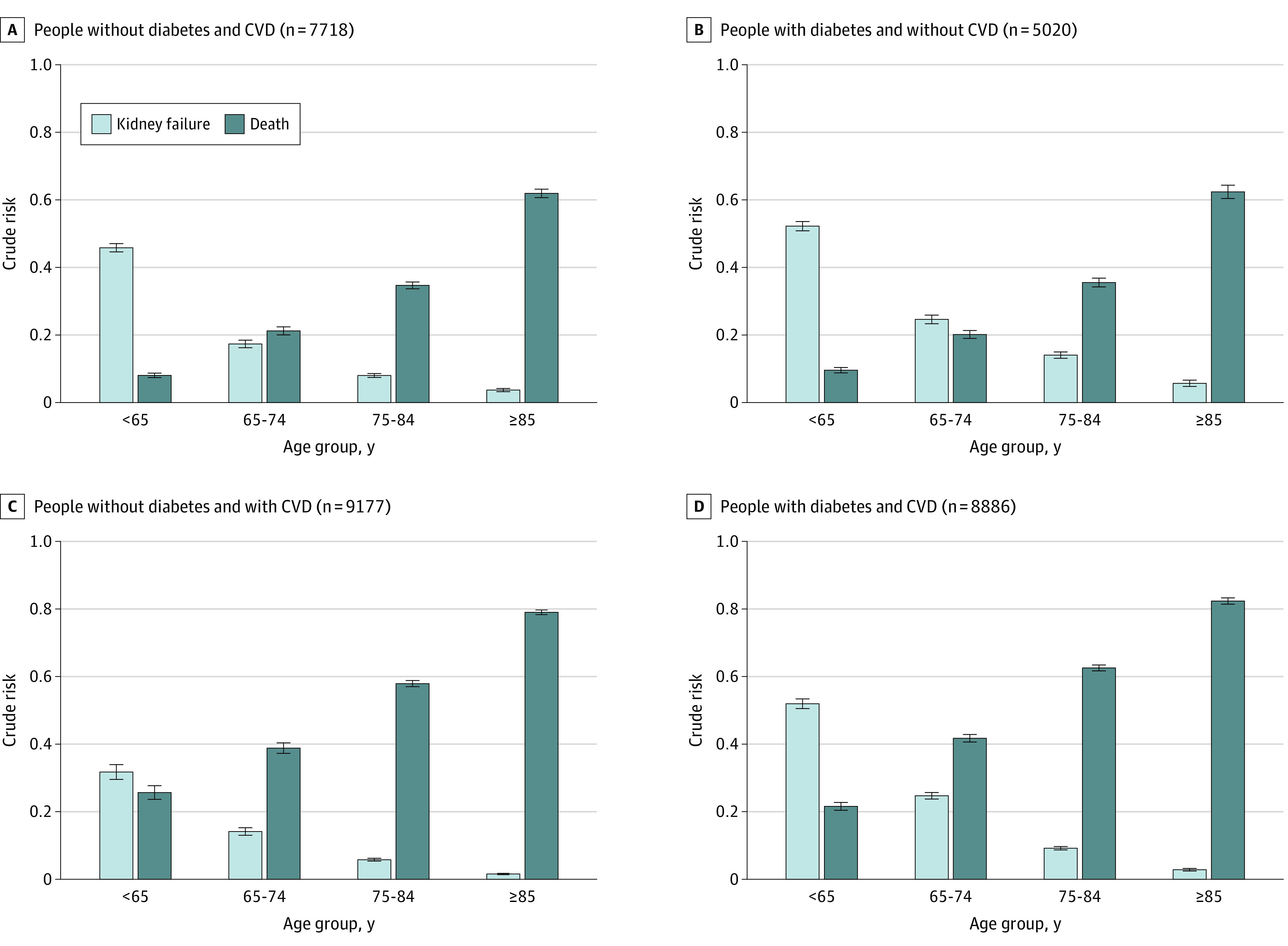
Stratified 5-Year Risks Cumulative incidence functions at 5 years by age, diabetes, and cardiovascular disease (CVD) are shown. For model-based 5-year risks by sex, see eFigures 8 and 9 in the Supplement. Error bars indicate 95% CIs.

The 5-year risk profiles across age groups were similar in 4 mutually exclusive categories of people with or without diabetes and with or without CVD (Figure 3). People with CVD had higher risk of death than kidney failure; we observed the opposite finding in those with diabetes. Although death was less likely than kidney failure among all 4 groups younger than 65 years regardless of comorbidity, the converse was true in 3 of 4 groups aged 65 to 74 years and all groups aged 75 years or older. Among people aged 75 to 84 years, the risk of death vs the risk of kidney failure ranged from 2.6-fold higher among those with diabetes but without CVD (0.36 [95% CI, 0.33-0.38] vs 0.14 [95% CI, 0.12-0.16]) to 9.6-fold higher among those with CVD but without diabetes (0.58 [95% CI, 0.56-0.6] vs 0.06 [95% CI, 0.05-0.07]). Among people aged 85 years or older, the risk of death exceeded the risk of kidney failure by a larger extent: 10.5-fold higher among those with diabetes but without CVD (0.62 [95% CI, 0.59-0.66] vs 0.06 [95% CI, 0.04-0.08]), and 39.5-fold higher among those without diabetes but with CVD (0.90 [95% CI, 0.78-0.81] vs 0.02 [95% CI, 0.01-0.02]).

When further subdivided into those aged 85 to 94 years and those aged 95 years or older, the risk of death vs the risk of kidney failure among those with CVD but without diabetes were 44.4-fold higher among those aged 85 to 94 years and 381-fold higher among those aged 95 years or older. Risk patterns across age groups were similar across categories defined by diabetes and CVD, albuminuria, or eGFR categories (eFigures 5 to 7 in the Supplement).

### Model-Based Risks and Sensitivity Analyses

Adjusted analyses (eTable 2 in the Supplement) were consistent with findings from crude and stratified analyses (eFigures 8 to 11 in the Supplement). Risk patterns were similar in women and men, although absolute risks were higher in men.

We obtained similar results in all sensitivity analyses (eFigures 12-20 in the Supplement). Model assumptions were met in all models.

## Discussion

The aging of the general population worldwide is viewed as one of the critical health challenges in the twenty-first century. As with many other age-related chronic health conditions, such as dementia, the prevalence of severe CKD has been shown to increase with age.^24^ Given that severe CKD is a precursor to kidney failure, this observation raises the possibility that population aging will expand the demand for renal replacement (dialysis or kidney transplant). This population-based cohort study found that the risk of death exceeded the risk of kidney failure in most people with stage IV CKD and that the risk of kidney failure decreased sharply with advancing age, regardless of comorbidity.

We believe that these findings provide some reassurance that, in contrast to the burden of other chronic diseases, the burden of kidney failure may not increase dramatically among those aged 85 years or older as the population ages. Although greater comorbidity was directly associated with both death and kidney failure, the risk of kidney failure decreased (and the risk of death increased) with older age, regardless of comorbidity. As opposed to CKD appearing earlier in life, CKD in older adults may result from multiple chronic-disease-causing mechanisms leading to accelerated frailty before kidney failure can ensue,^9^ although different comorbidities may have different associations with the CKD trajectory. For example, we found that people with CVD had a higher risk of death vs risk of kidney failure, whereas the opposite was observed in those with diabetes.

Taken together, these findings demonstrate that among older people with stage IV CKD, death was substantially more common than kidney failure, especially among those without diabetes, those with CVD, and those aged 75 years or older. These findings suggest that the projected increase in the coming decades in the number of older adults worldwide may be associated with a higher global burden of severe CKD but may not translate into a markedly increased prevalence of kidney failure in this population. Such findings should be reassuring for policymakers. Although population aging may mean that more resources are needed for the care and treatment of CKD and its associated comorbidities,^9^ the demand for costly renal replacement may be less than expected in older adults, especially given that many people who develop kidney failure as we defined it may choose to forgo renal replacement in favor of conservative care. If population aging were associated with increased prevalence of kidney failure, this association will be likely confined to people younger than 75 years and concentrated among those younger than 65 years who have a relatively high risk of kidney failure and who may maintain long life expectancy after developing kidney failure. Although the proportion of people aged 60 to 80 years is projected to increase over the next 3 decades, the relative magnitude of the increase (100%; from 825 million in 2017 to 1.7 billion in 2050) is much smaller than for those older than 80 years (>200%; from 137 to 425 million).^1^

We believe that this cohort study has clinical practice and research implications. First, the risk of death compared with the risk of kidney failure and the magnitude of each absolute risk are both important considerations when discussing prognosis with patients and their caregivers. In general, the risk of kidney failure exceeds the risk of death until older age in people with stage IV CKD who have diabetes and more severe albuminuria than in people without these risk factors, but the magnitude of each individual risk is far greater in the former than in the latter group. Second, instead of offering education about treatment options for kidney failure to all people with eGFR below a particular threshold, such education may be more relevant for low-risk, younger people and for high-risk people regardless of age. Third, a graphical representation of how the individual absolute risks of death and kidney failure vary over time or at prespecified times that uses a decision aid may better help patients and caregivers understand their values and goals.^6^ Such representations may help decision-makers understand the future need for renal replacement when considering data on the burden of kidney failure and the expected growth in the incidence of severe CKD in parallel with population aging. eFigures 21 and 22 in the Supplement present examples of such a decision aid.

Most national registries present the incidence and prevalence of kidney failure among older people in a single category (age >75 y), which may mask the decreasing risk of kidney failure among the older adult group.^25,26,27^ One exception is the ANZDATA (Australia and New Zealand Dialysis and Transplant) registry, which disaggregates these data for people aged 75 to 85 years and 85 years or older; consistent with our findings, this registry reports lower incident and prevalent use of renal replacement for kidney failure in those aged 85 years or older.^28^ The results of the current study are also consistent with those of previous studies that showed that advancing age, male sex, and comorbidity burden were important outcome factors in people with more severe^6,16^ and less severe^29^ nondialysis-dependent CKD. Consistent with studies that demonstrated that CKD progressed more slowly with older age,^14,30^ the present study suggests that the inverse association between age and the cause-specific hazard of kidney failure that we observed has a potentially causal association independent of the association between age and mortality.

However, this study differs from previous work in several ways and provides novel findings. We reported for the first time, to our knowledge, how risks and probabilities change with advancing age overall and across categories defined by common comorbidities. We used population-based data, included people with newly identified stage IV CKD at high risk of both death and kidney failure, and used eligibility criteria that minimize the inclusion of people with acute kidney injury or prevalent patients (as opposed to incident patients). Two previous studies included prevalent people with normal kidney function^13^ or stage III CKD.^14^ Other studies were based on selected cohorts of people referred for nephrology care^15^ or included people with stage V CKD (eGFR <15 mL/min/1.73 m^2^) who may already have kidney failure, and defined kidney failure as receipt of renal replacement.^14,15^ We defined kidney failure by receipt of renal replacement and by an objective measure of severe kidney impairment (eGFR <10 mL/min/1.73 m^2^), which means that these findings were not affected by the decision to choose conservative care.^13^

### Strengths and Limitations

This study has some strengths. First, we used population-based data from a geographically defined area served by a universal health care system. Second, we analyzed a large sample size and had an adequate follow-up period. Third, we used validated algorithms to ascertain the presence or absence of comorbidity, and we applied recommended methods in the setting of competing risks. Fourth, we used eligibility criteria to align sample characteristics to those of the target population with stage IV CKD. Fifth, we considered eGFR criteria to define kidney failure.

This study has several limitations. First, it relied on routinely collected data from people who accessed medical services from a single Canadian province, which had a low prevalence of people who self-identified as Black individuals. Second, we used the Chronic Kidney Disease Epidemiology Collaboration equation to estimate kidney function.^18^ Because this equation has not been as well validated in older people as other equations, it could have led to inaccurate eGFR for some people, although this inaccuracy should not have affected the findings regarding the relative risk of death vs kidney failure in the study population. Third, we had insufficient information to assess the association of eGFR decline before study entry with outcomes. Although the findings require validation in other settings, we do not believe that these limitations pose a serious threat to the validity of our conclusions.

## Conclusions

This cohort study found that among older people with stage IV CKD, death was substantially more common than kidney failure, especially among those with CVD and those older than 75 years. For policymakers, this finding suggests that the increase in the number of older adults (the fastest-growing age group) projected for the coming decades worldwide may not translate into a markedly increased prevalence of kidney failure in this population, unlike other age-related conditions such as dementia.

## Notes

Supplement. **eMethods** **eTable 1.** Codes for Identifying Dialysis or Transplantation **eTable 2.** Cause-Specific Hazard Ratios for Kidney Failure and Death **eFigure 1.** Derivation of Study Cohort **eFigure 2.** Crude Cumulative Incidence Functions by Age **eFigure 3.** Crude Risks of Kidney Failure With and Without Renal Replacement by Age **eFigure 4.** Crude 5-Year Risks of Kidney Failure With and Without Renal Replacement by Age **eFigure 5.** Stratified Probabilities **eFigure 6.** Crude Probabilities by Albuminuria **eFigure 7.** Crude Probabilities by eGFR **eFigure 8.** Model-Based Risks at Five Years (Women) **eFigure 9.** Model-Based Risks at Five Years (Men) **eFigure 10.** Model-Based Probabilities (Women) **eFigure 11.** Model-Based Probabilities (Men) **eFigure 12.** Crude Probabilities (Sensitivity Analysis) **eFigure 13.** Crude Probabilities (Sensitivity Analysis) **eFigure 14.** Crude Probabilities (Sensitivity Analysis) **eFigure 15.** Stratified Five-Year Risks (Sensitivity Analysis) **eFigure 16.** Stratified Five-Year Risks (Sensitivity Analysis) **eFigure 17.** Stratified Five-Year Risks (Sensitivity Analysis) **eFigure 18.** Stratified Probabilities (Sensitivity Analysis) **eFigure 19.** Stratified Probabilities (Sensitivity Analysis) **eFigure 20.** Stratified Probabilities (Sensitivity Analysis) **eFigure 21.** Clinical Scenarios 1 **eFigure 22.** Clinical Scenarios 2 **eReferences**
