# Supplementary material for: Association of Age With Risk of Kidney Failure in Adults With Stage IV Chronic Kidney Disease in Canada
Source: JAMA Netw Open. 2020 Sep 18;3(9):e2017150. doi: 10.1001/jamanetworkopen.2020.17150 (PMC7501537; doi:10.1001/jamanetworkopen.2020.17150)
Supplement: Supplement. — eMethods eTable 1. Codes for Identifying Dialysis or Transplantation eTable 2. Cause-Specific Hazard Ratios for Kidney Failure and Death eFigure 1. Derivation of Study Cohort eFigure 2. Crude Cumulative Incidence Functions by Age eFigure 3. Crude Risks of Kidney Failure With and Without Renal Replacement by Age eFigure 4. Crude 5-Year Risks of Kidney Failure With and Without Renal Replacement by Age eFigure 5. Stratified Probabilities eFigure 6. Crude Probabilities by Albuminuria eFigure 7. Crude Probabilities by eGFR eFigure 8. Model-Based Risks at Five Years (Women) eFigure 9. Model-Based Risks at Five Years (Men) eFigure 10. Model-Based Probabilities (Women) eFigure 11. Model-Based Probabilities (Men) eFigure 12. Crude Probabilities (Sensitivity Analysis) eFigure 13. Crude Probabilities (Sensitivity Analysis) eFigure 14. Crude Probabilities (Sensitivity Analysis) eFigure 15. Stratified Five-Year Risks (Sensitivity Analysis) eFigure 16. Stratified Five-Year Risks (Sensitivity Analysis) eFigure 17. Stratified Five-Year Risks (Sensitivity Analysis) eFigure 18. Stratified Probabilities (Sensitivity Analysis) eFigure 19. Stratified Probabilities (Sensitivity Analysis) eFigure 20. Stratified Probabilities (Sensitivity Analysis) eFigure 21. Clinical Scenarios 1 eFigure 22. Clinical Scenarios 2 eReferences [file jamanetwopen-e2017150-s001.pdf]

Supplementary Online Content

Ravani P, Quinn R, Fiocco M, et al. Association of age with risk of kidney failure in adults with stage IV chronic kidney disease in Canada. *JAMA Netw Open*. 2020;3(9):e2017150. doi:10.1001/jamanetworkopen.2020.17150

eMethods
eTable 1. Codes for Identifying Dialysis or Transplantation
eTable 2. Cause-Specific Hazard Ratios for Kidney Failure and Death
eFigure 1. Derivation of Study Cohort
eFigure 2. Crude Cumulative Incidence Functions by Age
eFigure 3. Crude Risks of Kidney Failure With and Without Renal Replacement by Age
eFigure 4. Crude 5-Year Risks of Kidney Failure With and Without Renal Replacement by Age
eFigure 5. Stratified Probabilities
eFigure 6. Crude Probabilities by Albuminuria
eFigure 7. Crude Probabilities by eGFR
eFigure 8. Model-Based Risks at Five Years (Women)
eFigure 9. Model-Based Risks at Five Years (Men)
eFigure 10. Model-Based Probabilities (Women)
eFigure 11. Model-Based Probabilities (Men)
eFigure 12. Crude Probabilities (Sensitivity Analysis)
eFigure 13. Crude Probabilities (Sensitivity Analysis)
eFigure 14. Crude Probabilities (Sensitivity Analysis)
eFigure 15. Stratified Five-Rear Risks (Sensitivity Analysis)
eFigure 16. Stratified Five-Rear Risks (Sensitivity Analysis)
eFigure 17. Stratified Five-Rear Risks (Sensitivity Analysis)
eFigure 18. Stratified Probabilities (Sensitivity Analysis)
eFigure 19. Stratified Probabilities (Sensitivity Analysis)
eFigure 20. Stratified Probabilities (Sensitivity Analysis)
eFigure 21. Clinical Scenarios 1
eFigure 22. Clinical Scenarios 2
eReferences

This supplementary material has been provided by the authors to give readers additional information about their work.

©2020 Ravani P et al. *JAMA Network Open*

eMethods

Study design and data sources

We conducted a population-based cohort study, using linked administrative and laboratory provincial data from Alberta, Canada (Alberta Health database).[1-5] The Alberta Health database contains information on demographic data, vital statistics, and diagnostic and procedural information for inpatient and outpatient physician services. All data were available from May 1, 2002 until March 31, 2017. Information about the receipt of renal replacement was available from April 1, 1994.

Over 99% of Alberta residents are registered with Alberta Health and have universal access to hospital care, laboratory testing and physician services. The institutional review boards at the Universities of Alberta (Pro00053469) and Calgary (REB16-1575) approved this study and waived the requirement for participants to provide consent.

Methods to calculate eGFR to define cohort entry and kidney failure

<u>Cohort entry</u>.

Moving average: Starting on the date of the first eGFR <30 ml/min/1.73 m^2, we determined the average eGFR over a period of >90 days provided that there were at least 2 outpatient measurements to calculate the mean.[6] Individuals with only one eGFR <30 ml/min/1.73 m^2 or with two values of eGFR <30 ml/min/1.73 m^2 recorded within 90 days were excluded. We used the minimum value of eGFR when there were multiple measurements on the same day. Participants met the criterion for stage 4 CKD when the average eGFR during this period was between 15 and <30 ml/min/1.73 m^2. We used the date of the last eGFR measurement included in the calculation of the average (index eGFR) to define cohort entry (index date).

<u>Kidney failure</u>.

Moving average (main analyses): Starting on the date of the first eGFR <10 ml/min/1.73 m^2, we determined the average of at least two inpatient or outpatient eGFR measurements made over a period of >90 days, using the minimum value of eGFR when there were multiple measurements on the same day. We used the date of the last eGFR measured during this period to define the event date. Individuals with only one eGFR <10 ml/min/1.73 m^2 or with two values of eGFR <10 ml/min/1.73 m^2 recorded within 90 days remained event-free (at risk); if they died after a single eGFR <10 ml/min/1.73 m^2 they were classified as dead.

Sustained reduction (sensitivity analyses): We defined sustained eGFR<10 ml/min/1.73 m^2 by the occurrence of ≥2 consecutive eGFR values <10 ml/min/1.73m^2 over a period of >90 days. We used the

©2020 Ravani P et al. *JAMA Network Open*

date of the last eGFR in the first episode of sustained eGFR measurements <10 ml/min/1.73 m^2 as the event date. Individuals with only one eGFR <10 ml/min/1.73 m^2 or with two values of eGFR <10 ml/min/1.73 m^2 recorded within 90 days remained event-free (at risk); if they died after a single eGFR <10 ml/min/1.73 m^2 they were classified as dead.

The threshold of <10 ml/min/1.73 m^2 allows for separation of the value defining cohort entry and the value defining the outcome. According to the Canadian registry of organ replacement, the mean eGFR at dialysis start has been between 9.5 and 10 ml/min/1.73 m^2 between 2002 and 2012.[7]

Independent variables, exposure and outcomes

We considered demographics and other characteristics that are associated with death or kidney failure: lower index eGFR, more severe albuminuria, diabetes and cardiovascular disease. The latter was defined as one or more of congestive heart failure, myocardial infarction, stroke or transient ischemic attack, or peripheral vascular disease (amputation or peripheral revascularization). We used validated coding algorithms applied to physician claims and hospitalization data to define comorbidities based on International Classification of Diseases, Ninth Revision, Clinical Modification (ICD-9-CM) and International Statistical Classification of Diseases, Tenth Revision (ICD-10).[8] We used the most recent albuminuria values (on or within the two years preceding the index date), with the following types of measurement in descending order of preference: albumin-to-creatinine ratio, protein-to-creatinine ratio, and urine dipstick. We categorized albuminuria as normal, moderate, severe, or unmeasured.[9]

Statistical analysis details

We used standard methods for qualitative data (frequencies) and quantitative data (mean/standard deviation) to summarize baseline information and competing risk analysis to estimate risks.[10]

<u>Cumulative incidence functions</u>. We estimated the crude and adjusted cumulative incidence functions of kidney failure and death without kidney failure and their 95% confidence intervals (CI) across age categories.[11] The cumulative incidence function (CIF) is the probability of failing from a specific cause k at time t. We obtained adjusted cumulative incidence functions for each event *indirectly* from a cause-specific hazard model of both kidney failure and death.[11] We used plots to summarize the crude and model-based cumulative incidence functions.[10,12]

<u>Model building and checking</u>. We used cause-specific Cox regression to model both cause-specific hazard functions simultaneously and derive cause-specific CIFs indirectly. We adjusted all models for

sex, eGFR, albuminuria category, diabetes and presence of cardiovascular disease, and tested all possible first-order interactions among these variables. We used fractional polynomials, spline functions and martingale residuals analyses to assess the form of the relationship between continuous covariates and outcome. We used residual analyses to identify deviations from proportionality assumption, influential observations and outliers, and for assessment of goodness-of-fit. During model building we checked that results were consistent across study time.
We used R (https://www.r-project.org/) for all analyses.[10-15]

Sensitivity analyses. We tested consistency of findings in several sensitivity analyses First, we defined kidney failure as initiation of renal replacement or occurrence of sustained eGFR<10 ml/min/1.73 m^2. Second, we defined kidney failure solely by initiation of renal replacement, defined as receipt of a kidney transplant or registration in the provincial database of chronic dialysis. Third, we restricted the analyses to complete cases for albuminuria (excluding those with unmeasured values).

eTable 1. Codes for Identifying Dialysis or Transplantation

1) **Physician claims**: Canadian Classification of Diagnostic, Therapeutic, and Surgical Procedures codes

Codes	Code description
For dialysis	
13.99A	Hemodialysis treatment, unstable patient
13.99B	Hemodialysis treatment, stable patient
13.99C	Assessment and management of an unstable patient with acute/chronic renal failure treated by peritoneal dialysis
13.99D	Assessment and management of a stable patient with chronic renal failure treated by peritoneal dialysis
13.99O	Management of dialysis patients on home dialysis or receiving treatment in a remote hemodialysis unit (per week)
13.99OA	Management of patient on hemodialysis or peritoneal dialysis (per week)
13.99AB	Dialysis therapy, any modality, in the intensive care unit
For transplantation	
67.5	Transplant of kidney
67.59	Other kidney transplantation
67.59A	Renal transplantation (homo, hetero, auto)

2) **Hospitalizations**: Canadian Classification of Health Intervention codes

Codes	Code description
For transplantation	
1.PC.85.^^	Transplant, kidney
1.PC.85.LA-XX-J	Using living donor (allogenic or syngeneic) kidney
1.PC.85.LA-XX-K	Using deceased donor kidney
1.OK.85.XU-XX-K	Transplant, pancreas with duodenum and kidney with exocrine drainage via bladder [e.g. donor duodenum is grafted to bladder: duodenocystostomy]
1.OK.85.XV-XX-K	Transplant, pancreas with duodenum and kidney with exocrine drainage via intestine with homograft [e.g. donor duodenum is grafted to bowel]

eTable 2. Cause-Specific Hazard Ratios for Kidney Failure and Death

Kidney failure	**HR**	**LCI**	**UCI**
Age (10 years)	0.54	0.51	0.57
Age^2 (10 $years^2$)	0.95	0.94	0.96
eGFR (5 ml/min/1.73 m^2)	0.49	0.45	0.54
Age X eGFR (*)	0.96	0.94	0.98
$eGFR^2$ ([5 vml/min/1.73 m^2] ^{2})	1.03	0.99	1.07
Sex (male vs female)	1.79	1.61	1.99
Diabetes (present vs absent)	1.59	1.45	1.75
CVD (present vs absent)	0.81	0.73	0.91
A0 vs A1	1.94	1.71	2.21
A2 vs A1	1.68	1.53	1.84
A3 vs A1	4.29	3.96	4.65
Male X diabetes	0.74	0.66	0.82
Diabetes X CVD	1.22	1.08	1.36
Age X male	1.06	1.03	1.1
Age X CVD	0.91	0.87	0.95
Death			
Age (10 years)	1.96	1.89	2.03
Age^2 (10 $years^2$)	1.05	1.04	1.06
eGFR (5 ml/min/1.73 m^2)	0.87	0.84	0.9
Age X eGFR (*)	0.96	0.94	0.99
Sex (male vs female)	0.94	0.9	0.99
Diabetes (present vs absent)	1.27	1.21	1.32
CVD (present vs absent)	1.07	1	1.15
A0 vs A1	1.84	1.75	1.92
A2 vs A1	1.38	1.31	1.45
A3 vs A1	1.17	1.13	1.22
Male X diabetes	1.3	1.24	1.36
Diabetes X CVD	0.95	0.89	1.01
Age X male	1.1	1.02	1.18
Age X CVD	1.1	1.06	1.14
eGFR X diabetes	0.87	0.84	0.9

Legend: Age centered on 80 years (median = 79.6; mean = 76.8), effect per 10 years of age; eGFR estimated glomerular filtration rate centered on 27 ml/min/1.73 m^2 (median = 27.2; mean = 26.2), effect per 5 ml/min/1.73 m^2; Age X eGFR (*): 10 years of age by 5 ml/min/1.73 m^2 of eGFR; CVD: cardiovascular disease; A0 indicates missing albuminuria value; A1-A3: see table 1 for albuminuria categories. N of events: kidney failure = 5,511 (renal replacement = 4,758); death = 16,285. HR indicates hazard ratio; LCI indicates the lower bound of the 95% confidence interval; UCI indicates the upper bound of the 95% confidence interval.

eFigure 1. Derivation of Study Cohort

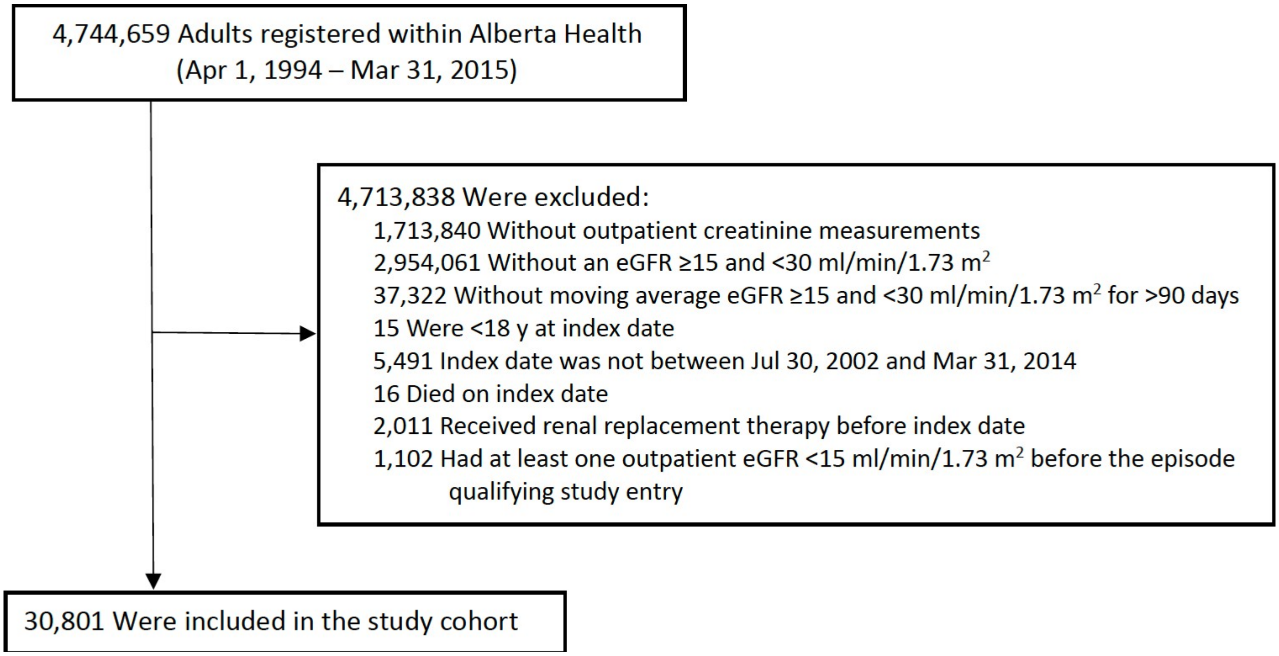

eFigure 2. Crude Cumulative Incidence Functions by Age

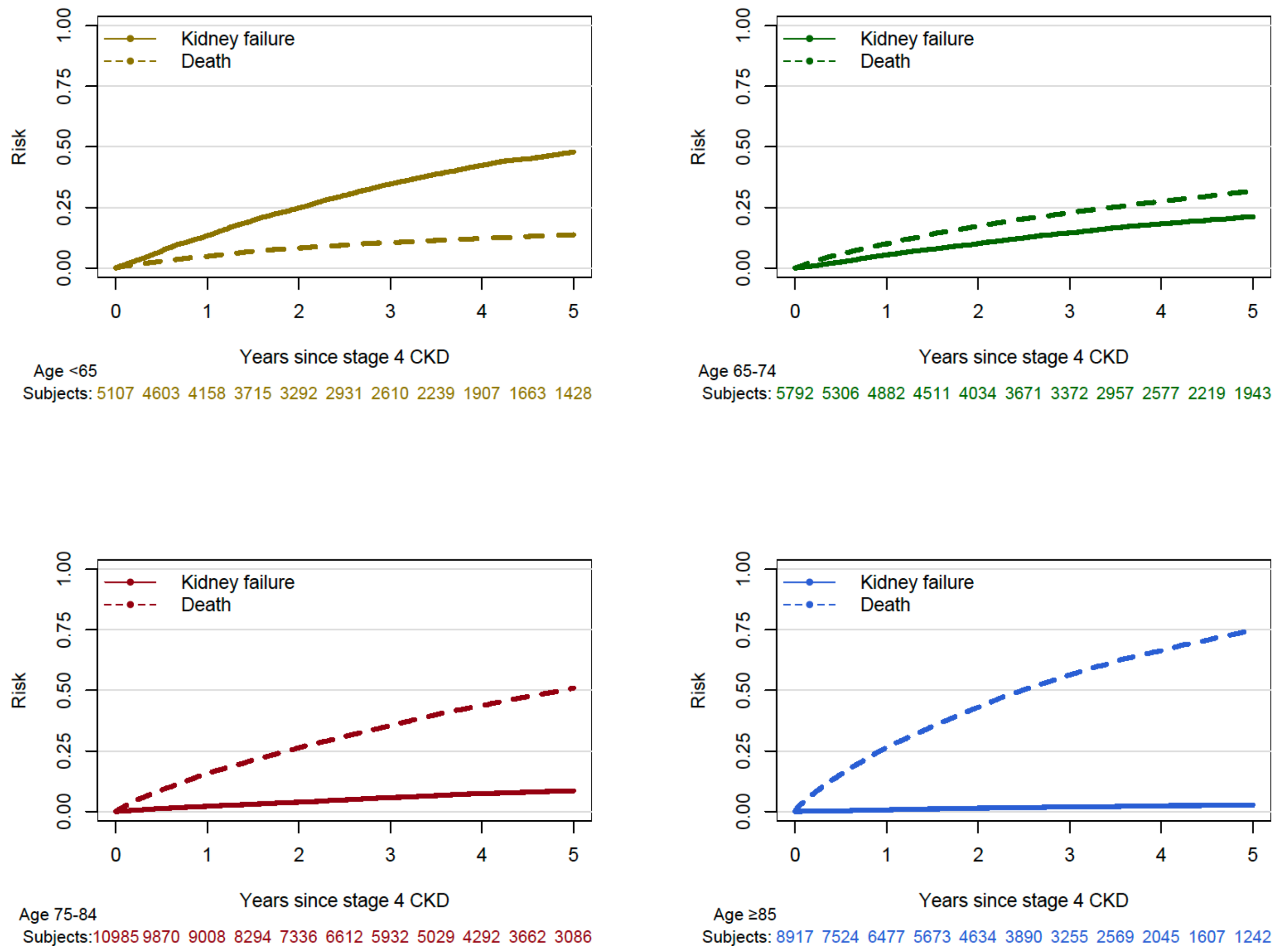

Legend: Overlaid cumulative incidence functions (crude risks over time) across age categories (in years).

©2020 Ravani P et al. *JAMA Network Open*

eFigure 3. Crude Risks of Kidney Failure With and Without Renal Replacement by Age

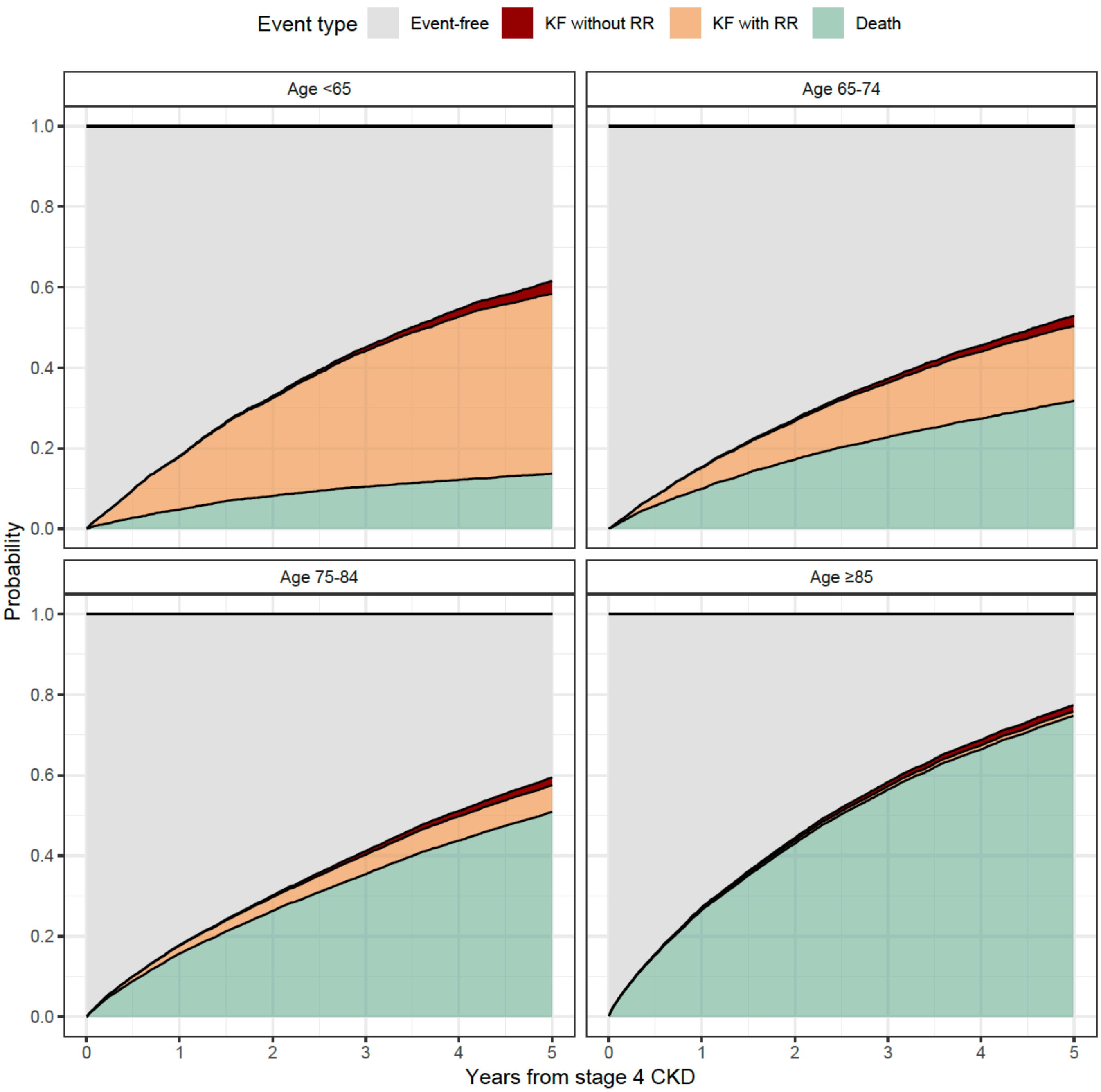

Legend: Stacked cumulative incidence functions by age in years (crude probabilities over time). KF without RR indicates kidney failure without renal replacement; KF with RR indicates kidney failure with renal replacement.

©2020 Ravani P et al. *JAMA Network Open*

eFigure 4. Crude 5-Year Risks of Kidney Failure With and Without Renal Replacement by Age

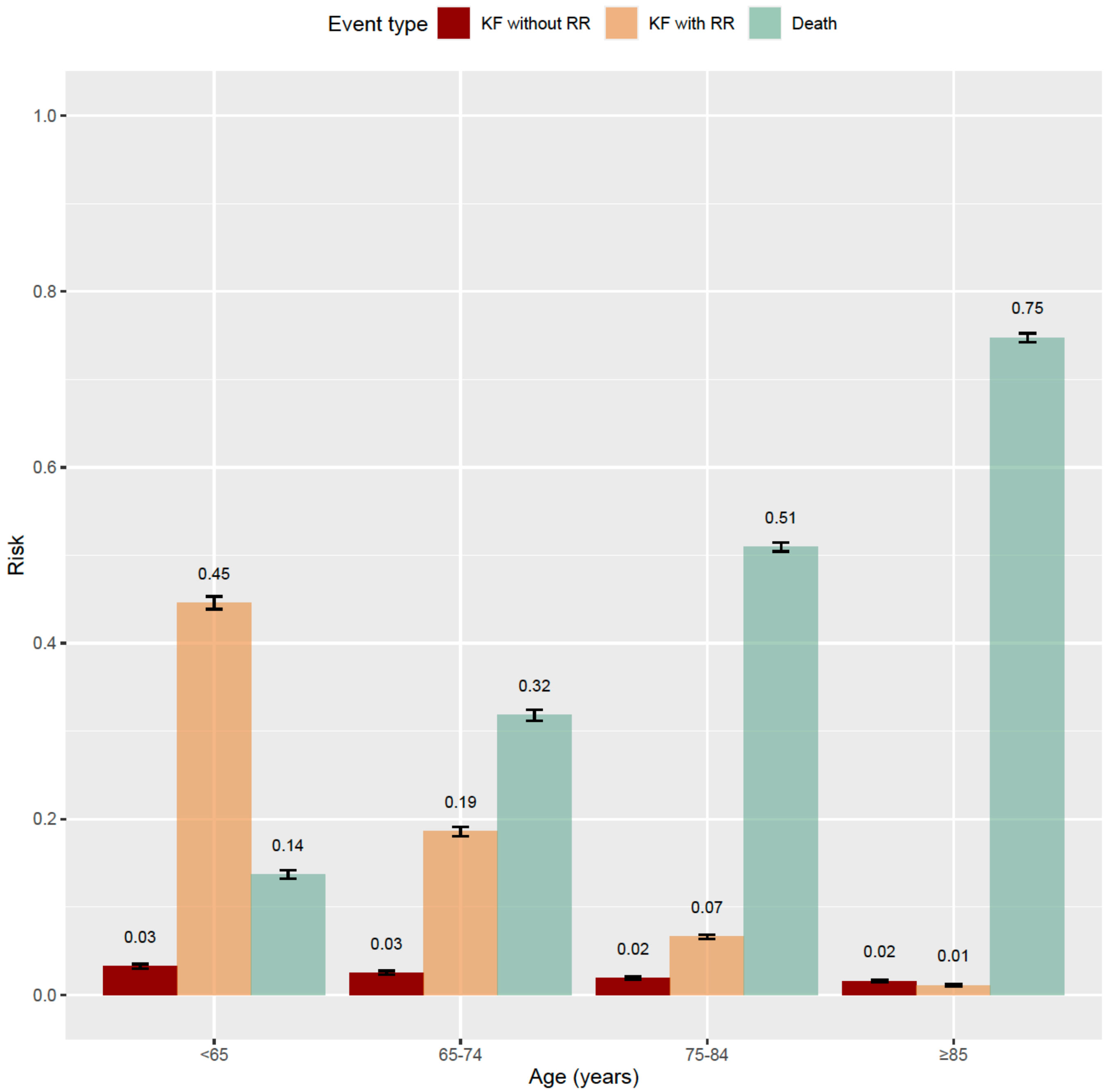

Legend: Cumulative incidence functions at 5 years by age.

©2020 Ravani P et al. *JAMA Network Open*

eFigure 5. Stratified Probabilities

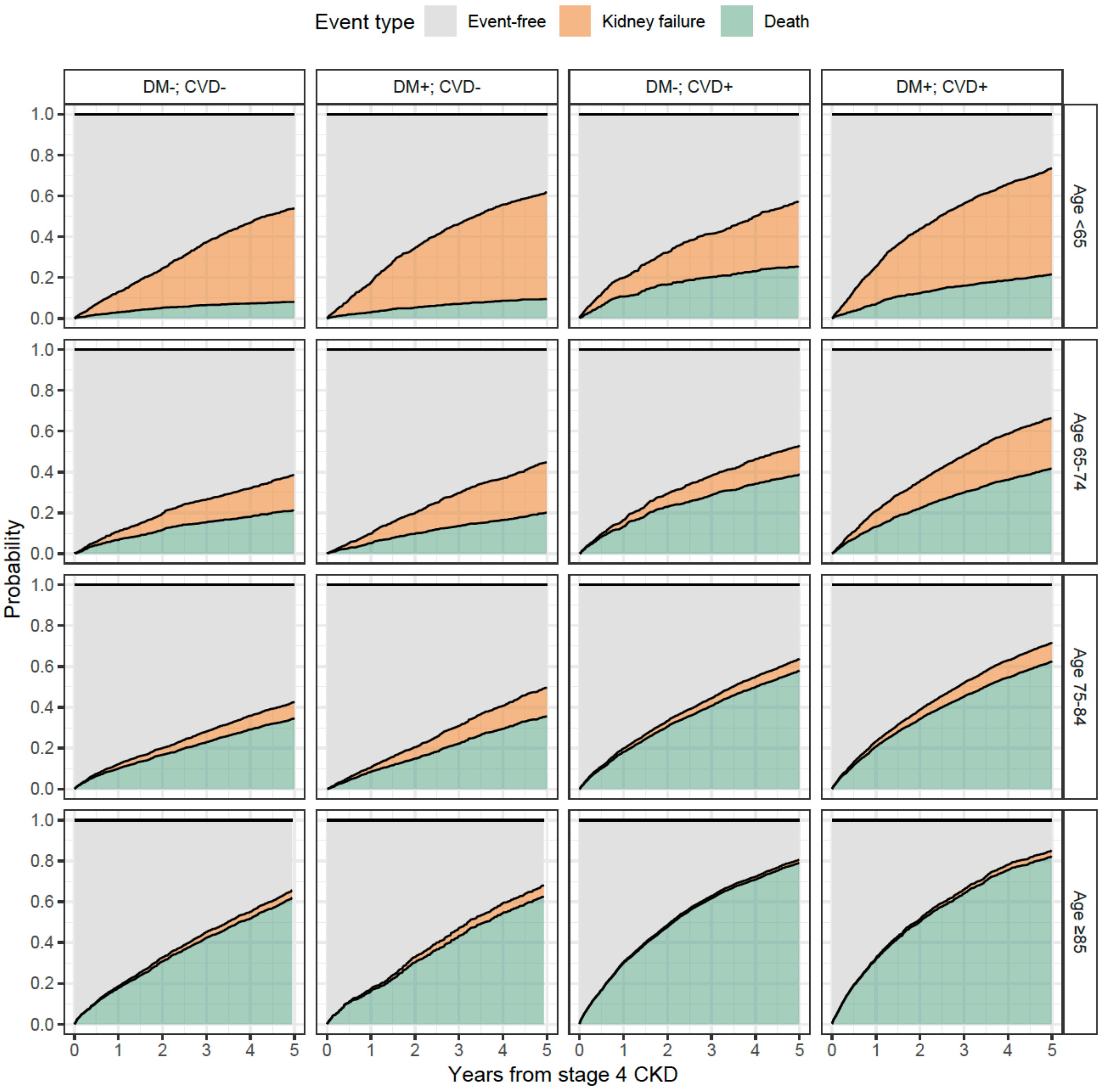

Legend: Stacked cumulative incidence functions (crude probabilities over time) stratified by age, diabetes (DM) and cardiovascular disease (CV). DM-/+ indicate absence (-) or presence of diabetes (DM); CV-/+ indicate absence (-) or presence of cardiovascular disease (CV). For model-based probabilities in women and men see Supplement eFigures 10-11.

©2020 Ravani P et al. *JAMA Network Open*

eFigure 6. Crude Probabilities by Albuminuria

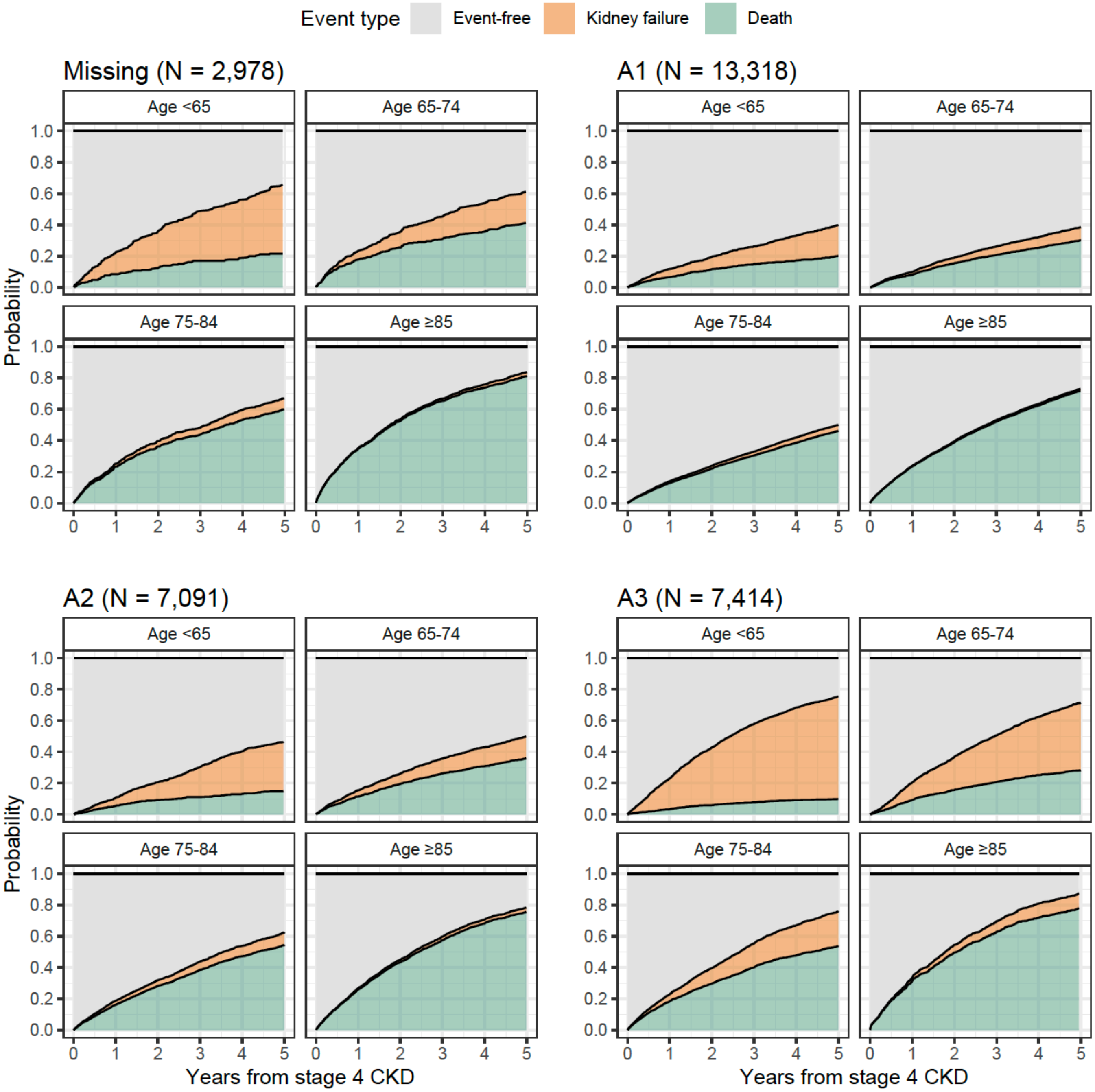

Legend: Stacked cumulative incidence functions (crude probabilities over time); A1, A2, and A3 are categories of proteinuria, which we calculated according to the KDIGO recommendations:
A1 = albuminuria <30 mg/24 hrs, or proteinuria <150 mg/24 hrs, or albumin-creatinine ratio [ACR] <3 mg/mmol (<30 mg/g), or protein-creatinine ratio [PCR] <15 mg/mmol (<150 mg/g), or protein reagent strip negative to trace; A2 = albuminuria 30-300 mg/24 hrs, or proteinuria 150-500 mg/24 hrs, or albumin-creatinine ratio [ACR] 3-30 mg/mmol (30-300 mg/g), or protein-creatinine ratio [PCR] 15-50 mg/mmol (150-500 mg/g), or protein reagent strip trace to +; A3 = albuminuria >300 mg/24 hrs, or proteinuria >500 mg/24 hrs, or albumin-creatinine ratio [ACR] >30 mg/mmol (>300 mg/g), or protein-creatinine ratio [PCR] >50 mg/mmol (>500 mg/g), or protein reagent strip + or greater.

©2020 Ravani P et al. *JAMA Network Open*

eFigure 7. Crude Probabilities by eGFR

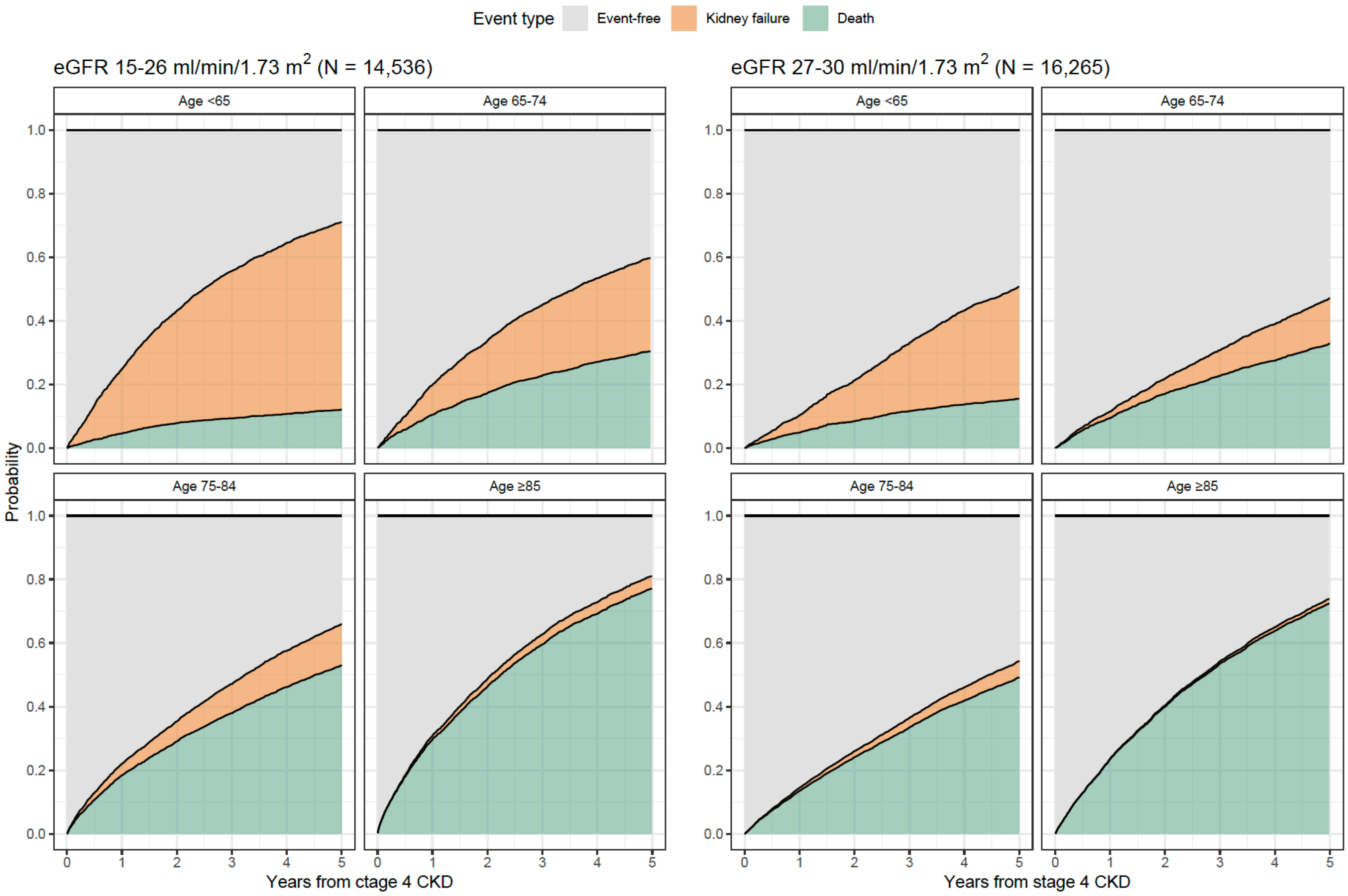

Legend: Stacked cumulative incidence functions (crude probabilities over time); eGFR indicates estimated glomerular filtration rate.

©2020 Ravani P et al. *JAMA Network Open*

eFigure 8. Model-Based Risks at Five Years (Women)

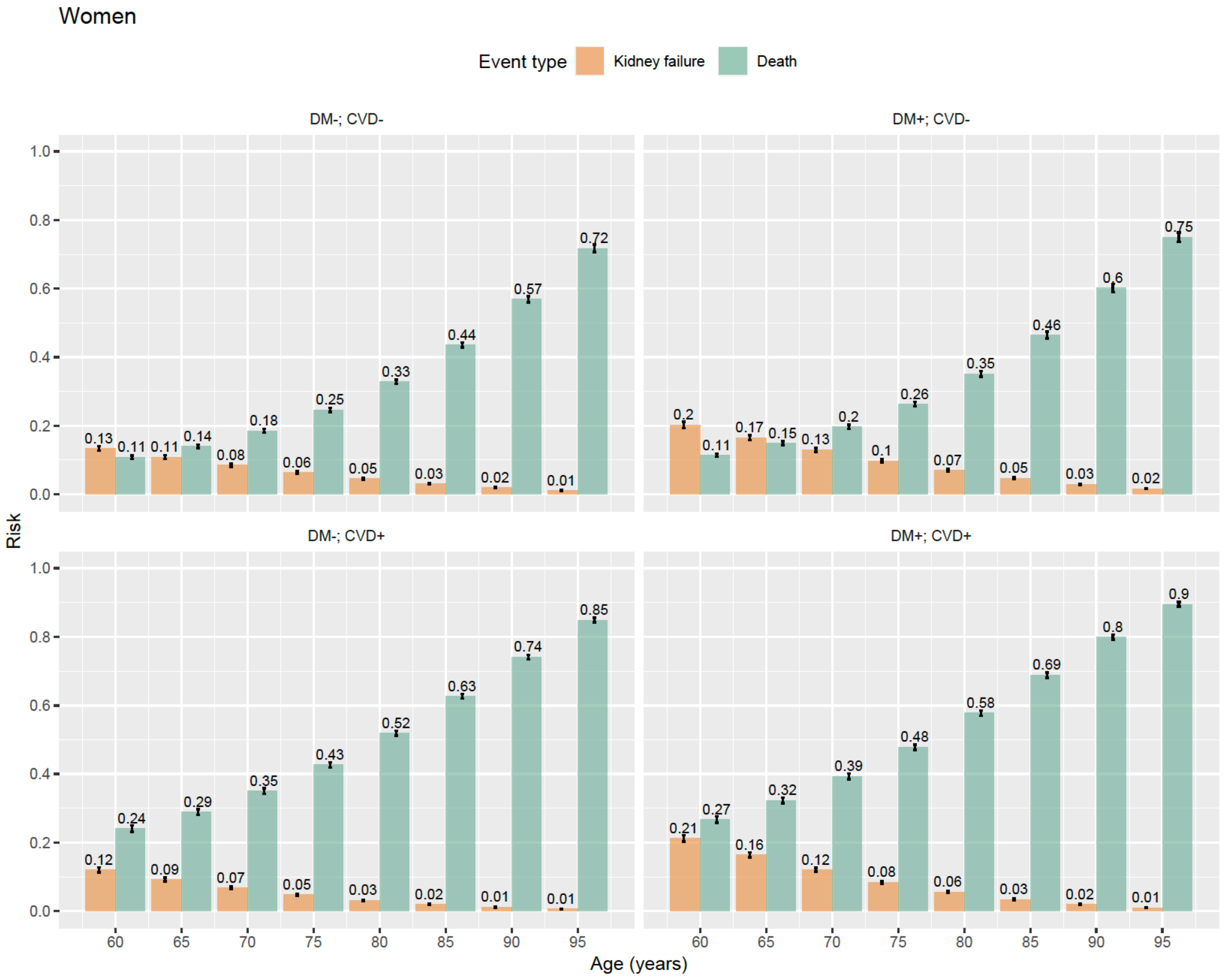

Legend: Model-based risks (eTable 2) in women for ages 60 to 95, assuming absence of albuminuria (A1) and an eGFR of 25 ml/min/m^2. DM-/+ indicate absence (-) or presence of diabetes (DM); CV-/+ indicate absence (-) or presence of cardiovascular disease (CV).

©2020 Ravani P et al. *JAMA Network Open*

eFigure 9. Model-Based Risks at Five Years (Men)

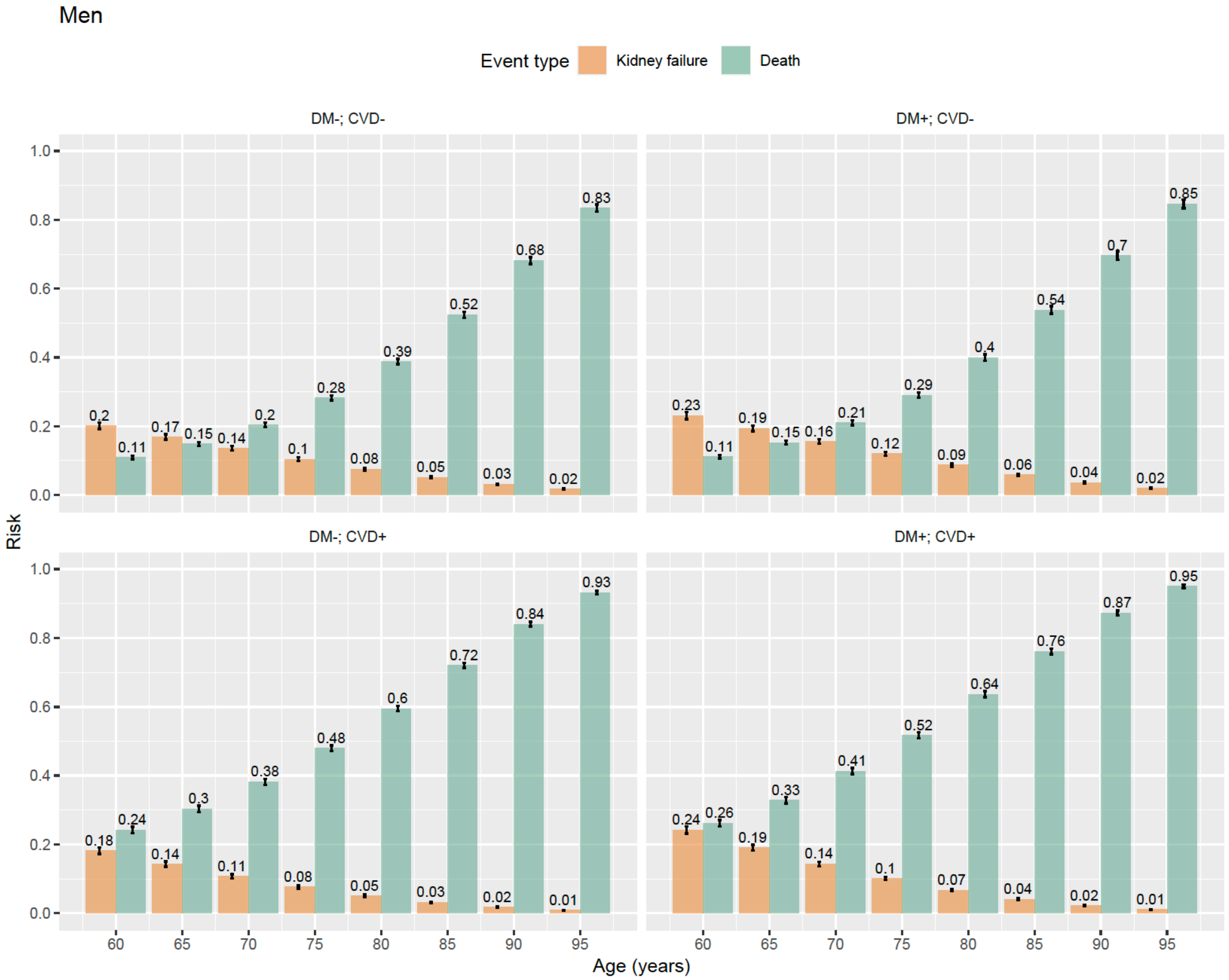

Legend: Model-based risks (eTable 2) in men for ages 60 to 95, assuming absence of albuminuria (A1) and an eGFR of 25 ml/min/m^2. DM-/+ indicate absence (-) or presence of diabetes (DM); CV-/+ indicate absence (-) or presence of cardiovascular disease (CV).

©2020 Ravani P et al. *JAMA Network Open*

eFigure 10. Model-Based Probabilities (Women)

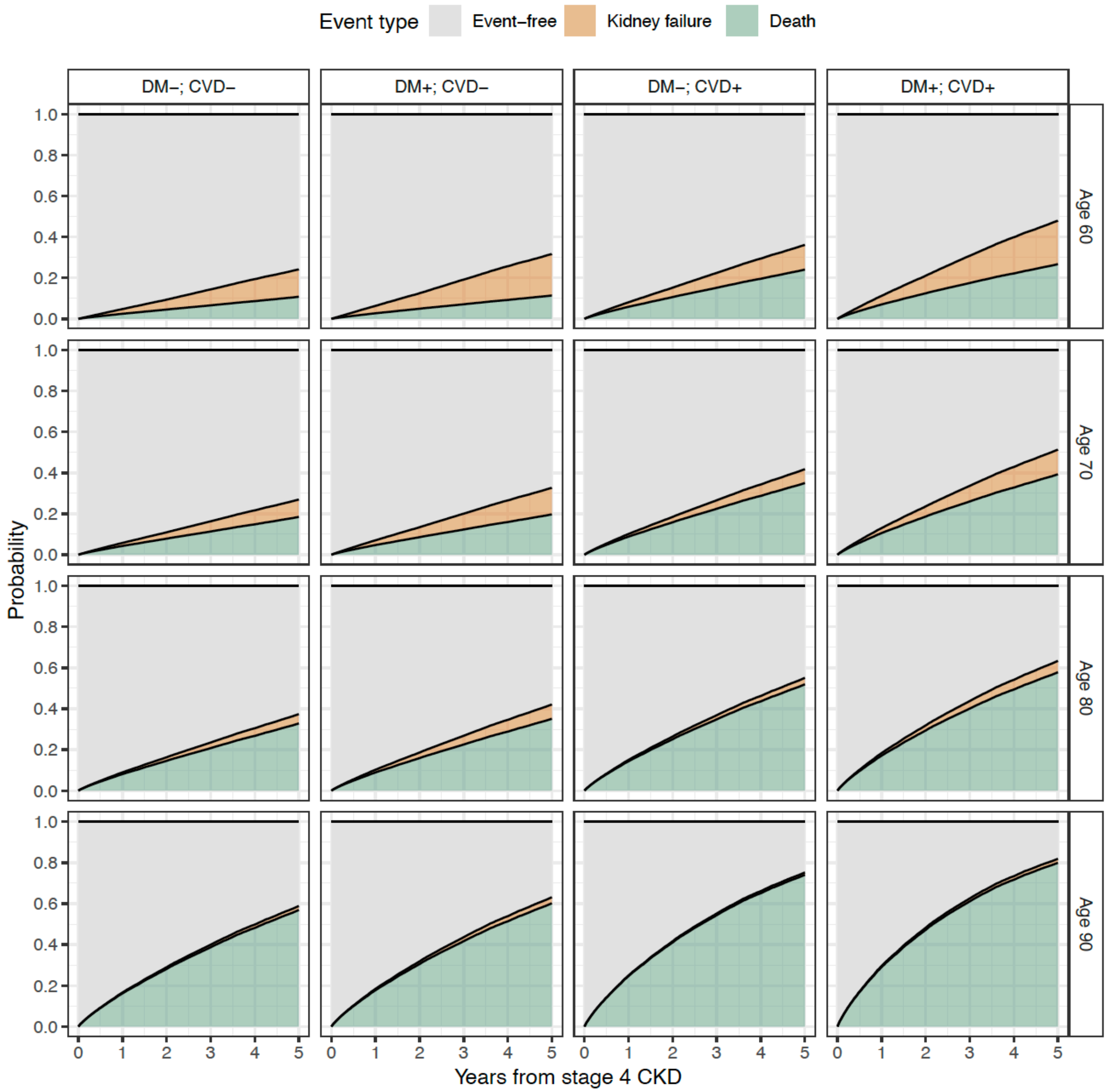

Legend: Stacked cumulative incidence functions (model-based probabilities over time; eTable 2). Probabilities are reported for ages 60, 70, 80 and 90 by absence/presence of diabetes (DM-/+) and cardiovascular disease (CV-/+) in women without albuminuria (A1) and with an eGFR of 25 ml/min/m^2.

©2020 Ravani P et al. *JAMA Network Open*

eFigure 11. Model-Based Probabilities (Men)

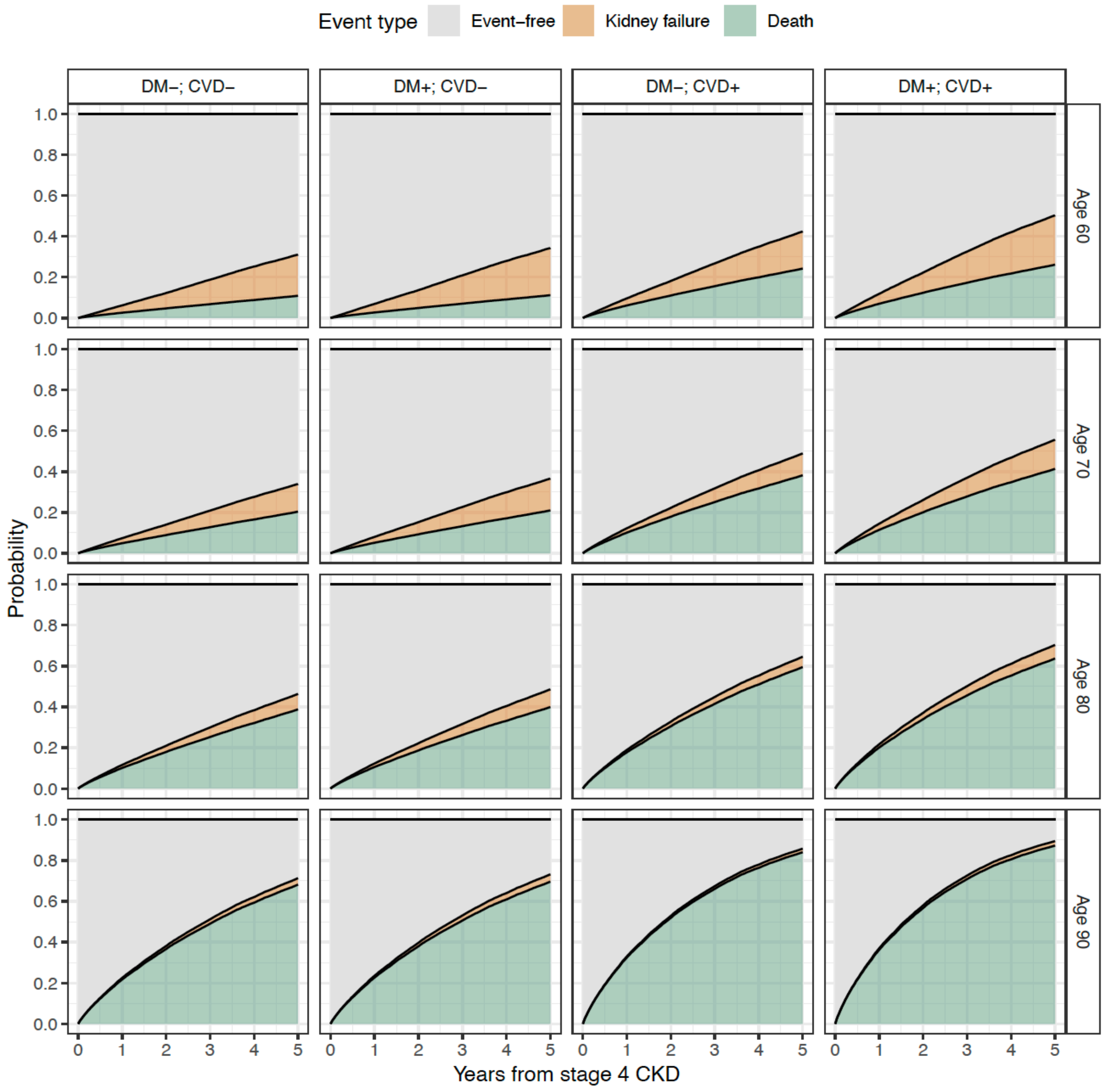

Legend: Stacked cumulative incidence functions (model-based probabilities over time; eTable 2). Probabilities are reported for ages 60, 70, 80 and 90 by absence/presence of diabetes (DM-/+) and cardiovascular disease (CV-/+) in men without albuminuria (A1) and with an eGFR of 25 ml/min/m^2.

©2020 Ravani P et al. *JAMA Network Open*

eFigure 12. Crude Probabilities (Sensitivity Analysis)

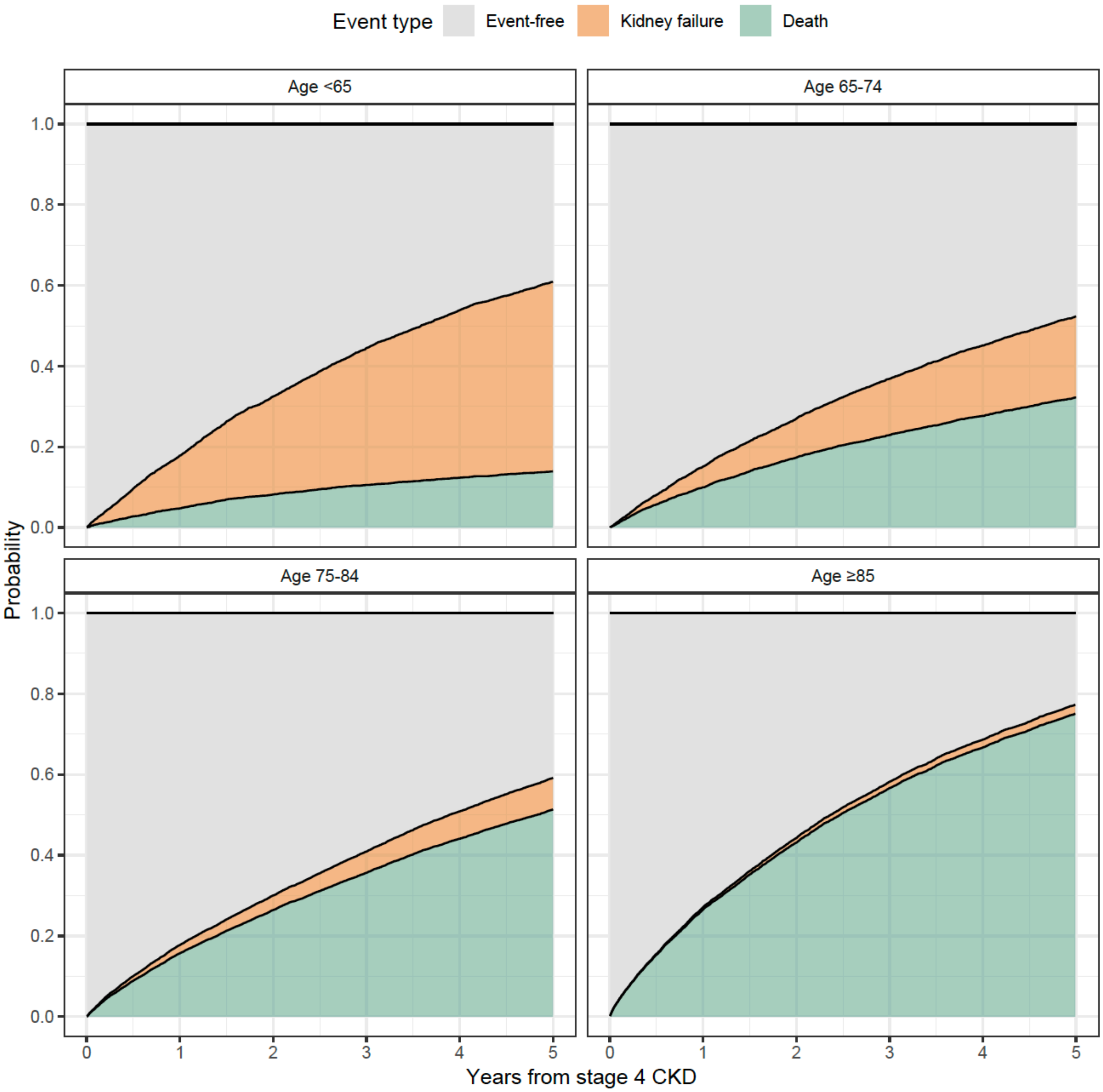

Legend: Stacked cumulative incidence functions by age (crude probabilities over time). In this analysis kidney failure was defined as initiation of renal replacement therapy or sustained eGFR<10 ml/min/1.73 m^2 (occurrence of ≥2 consecutive eGFR values <10 ml/min/1.73m^2 over a period of >90 days). N of events: kidney failure = 5,253 (renal replacement = 4,758); death = 16,472.

©2020 Ravani P et al. *JAMA Network Open*

eFigure 13. Crude Probabilities (Sensitivity Analysis)

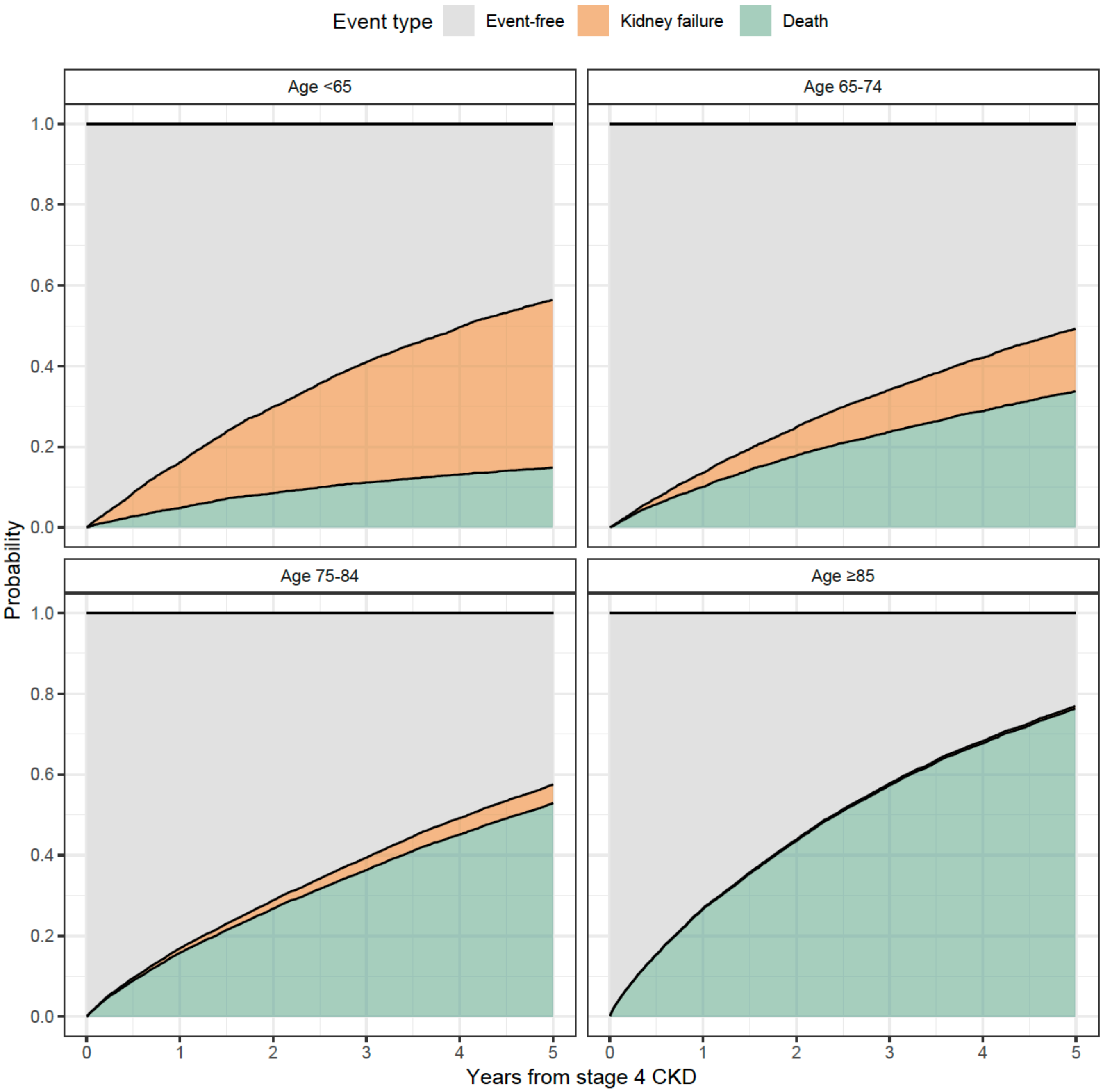

Legend: Stacked cumulative incidence functions by age (crude probabilities over time). In this analysis kidney failure was defined as initiation of renal replacement therapy (receipt of a kidney transplant or registration in the provincial database of chronic dialysis). N of events: kidney failure = 3,940; death = 16,826.

©2020 Ravani P et al. *JAMA Network Open*

eFigure 14. Crude Probabilities (Sensitivity Analysis)

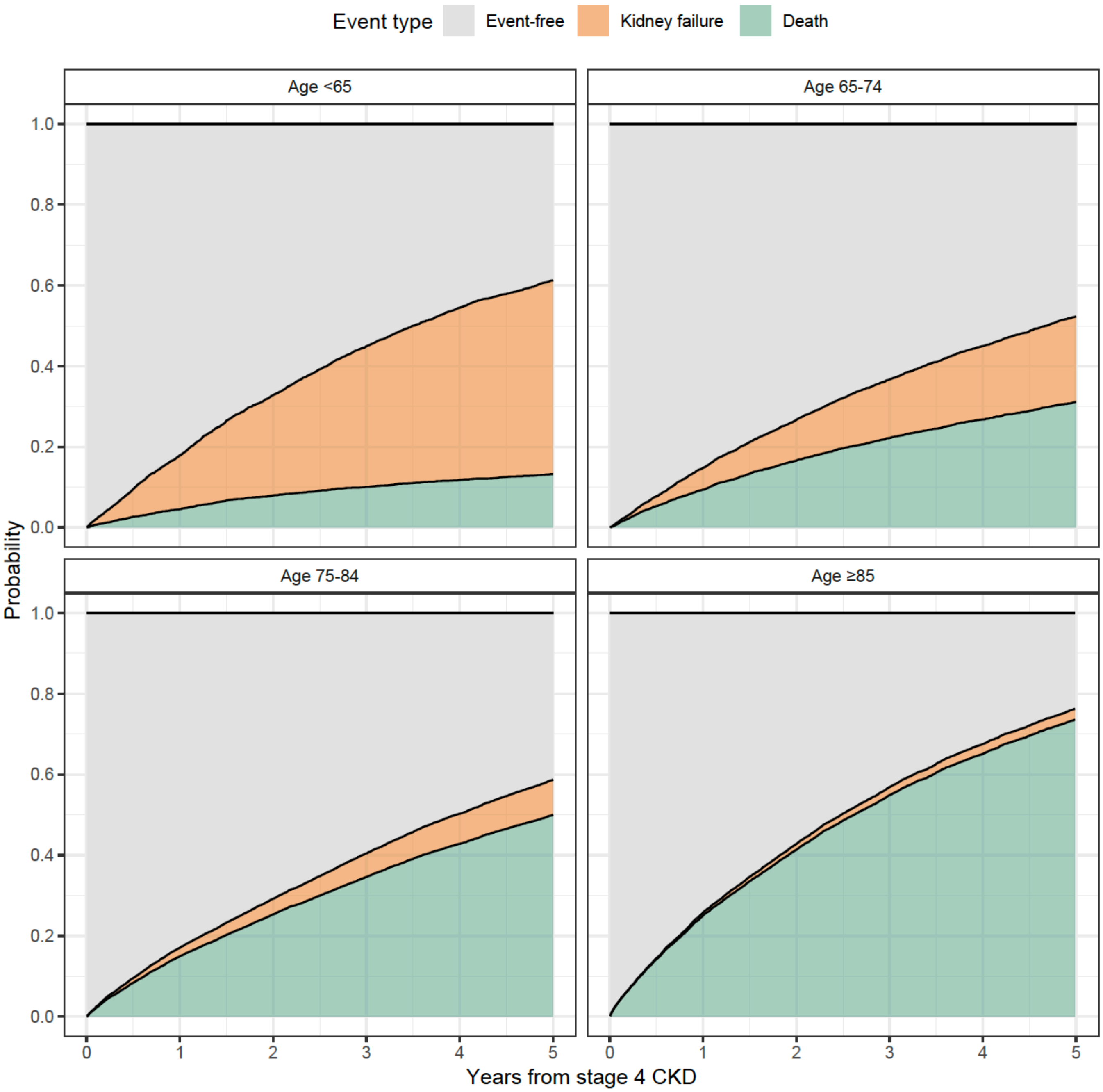

Legend: Stacked cumulative incidence functions by age (crude probabilities over time). This analysis was restricted to complete cases for albuminuria. N of events: kidney failure = 5,181; death = 14,247.

©2020 Ravani P et al. *JAMA Network Open*

eFigure 15. Stratified Five-Year Risks (Sensitivity Analysis)

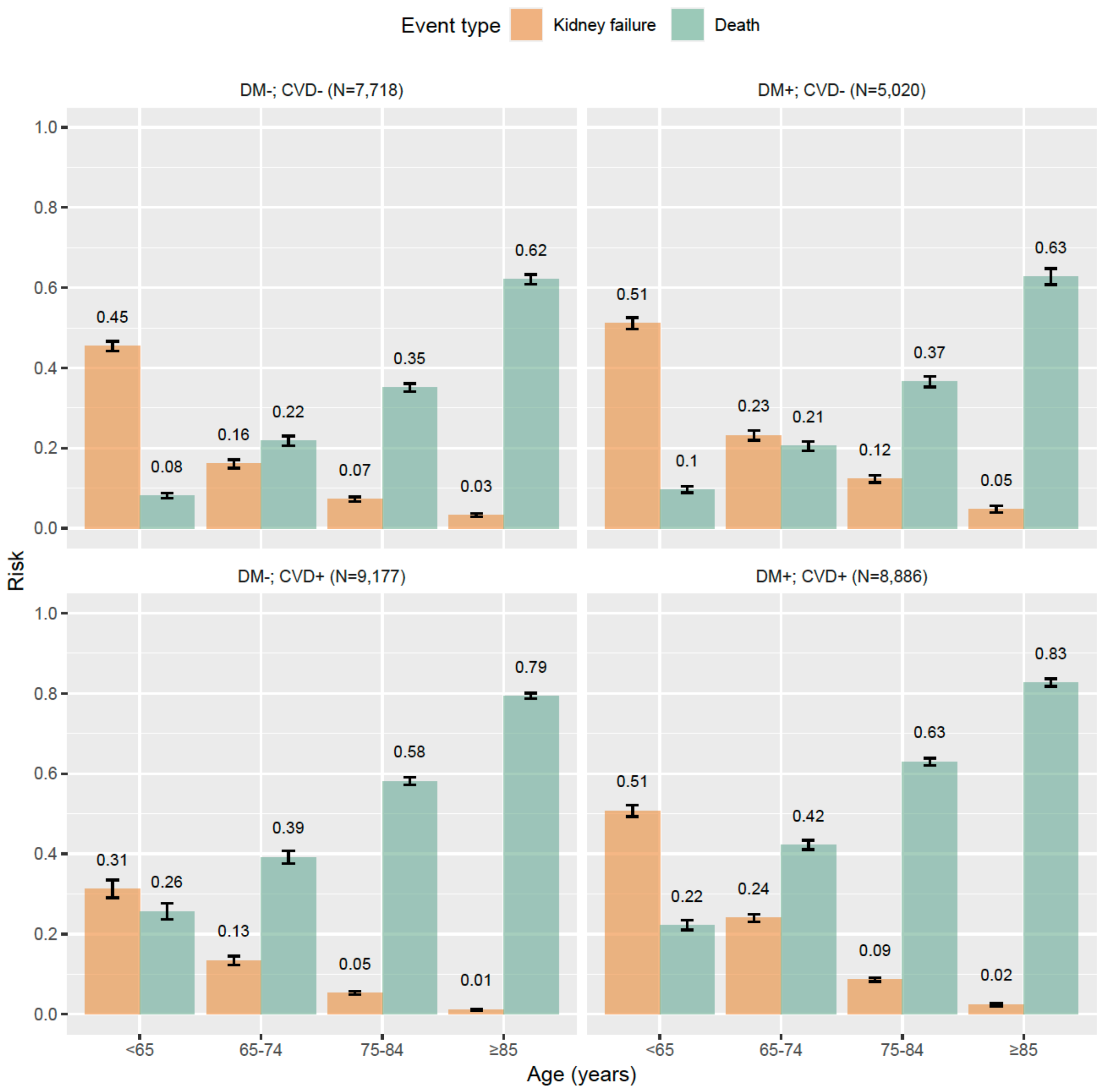

Legend: Cumulative incidence functions at 5 years by age, diabetes and cardiovascular disease (crude 5-year risks). DM-/+ indicate absence (-) or presence of diabetes (DM); CV-/+ indicate absence (-) or presence of cardiovascular disease (CV). In this analysis kidney failure was defined as initiation of renal replacement therapy or sustained eGFR<10 ml/min/1.73 m^2 (occurrence of ≥2 consecutive eGFR values <10 ml/min/1.73m^2 over a period of >90 days).

eFigure 16. Stratified Five-Year Risks (Sensitivity Analysis)

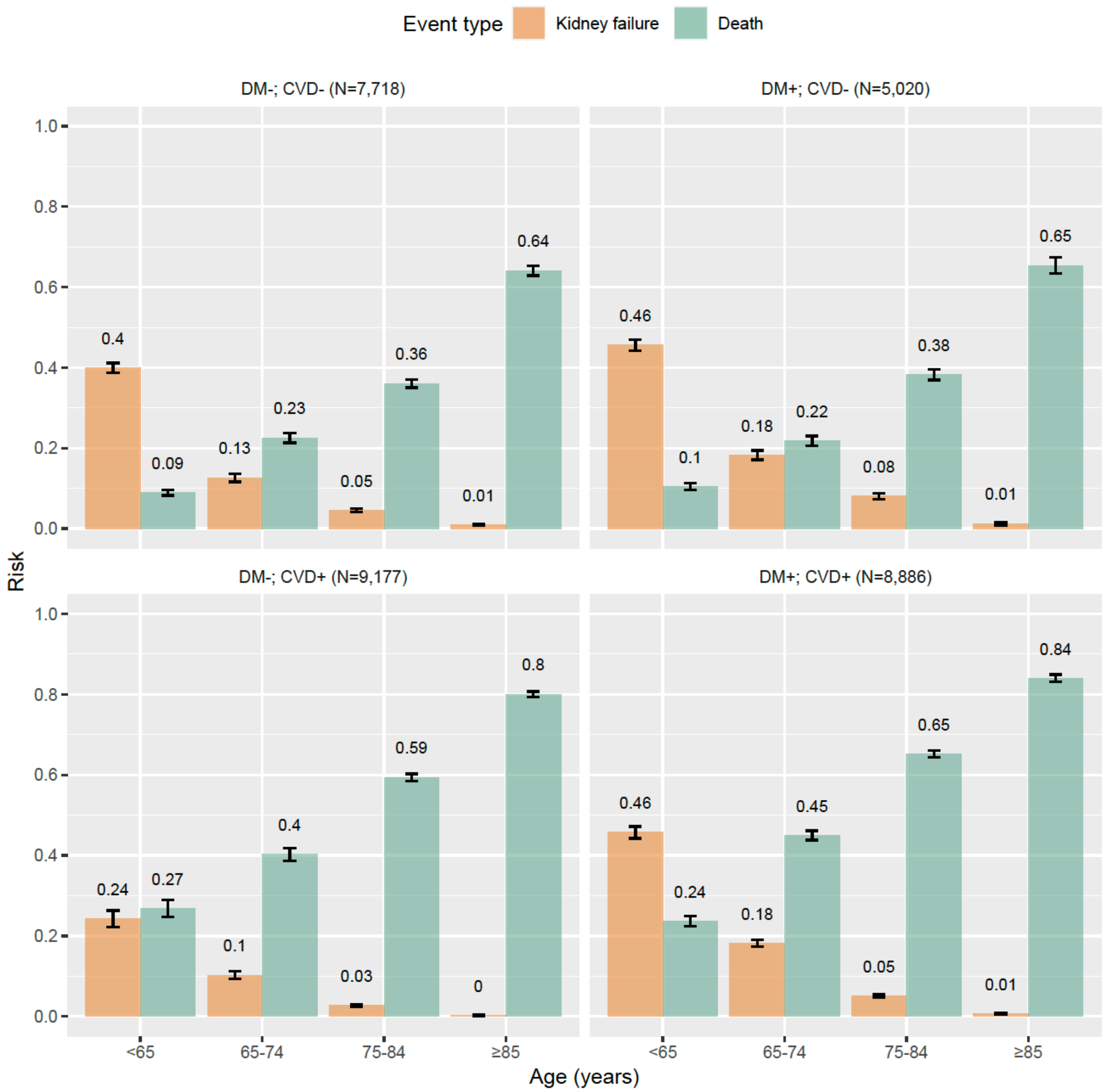

Legend: Cumulative incidence functions at 5 years by age, diabetes and cardiovascular disease (crude 5-year risks). DM-/+ indicate absence (-) or presence of diabetes (DM); CV-/+ indicate absence (-) or presence of cardiovascular disease (CV). In this analysis kidney failure was defined as initiation of renal replacement therapy (receipt of a kidney transplant or registration in the provincial database of chronic dialysis).

©2020 Ravani P et al. *JAMA Network Open*

eFigure 17. Stratified Five-Year Risks (Sensitivity Analysis)

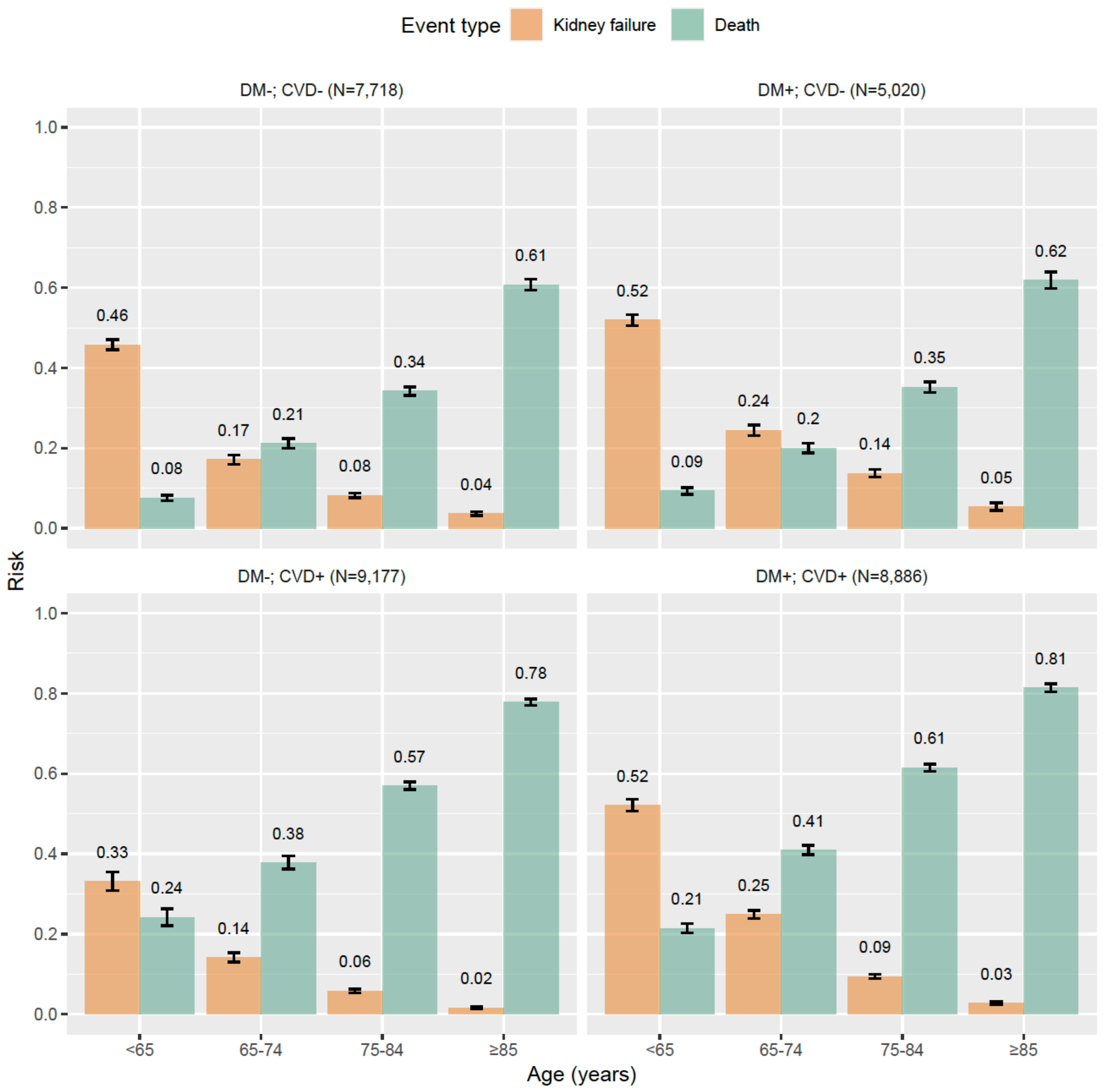

Legend: Cumulative incidence functions at 5 years by age, diabetes and cardiovascular disease (crude 5-year risks). DM-/+ indicate absence (-) or presence of diabetes (DM); CV-/+ indicate absence (-) or presence of cardiovascular disease (CV). This analysis was restricted to complete cases for albuminuria.

©2020 Ravani P et al. *JAMA Network Open*

eFigure 18. Stratified Probabilities (Sensitivity Analysis)

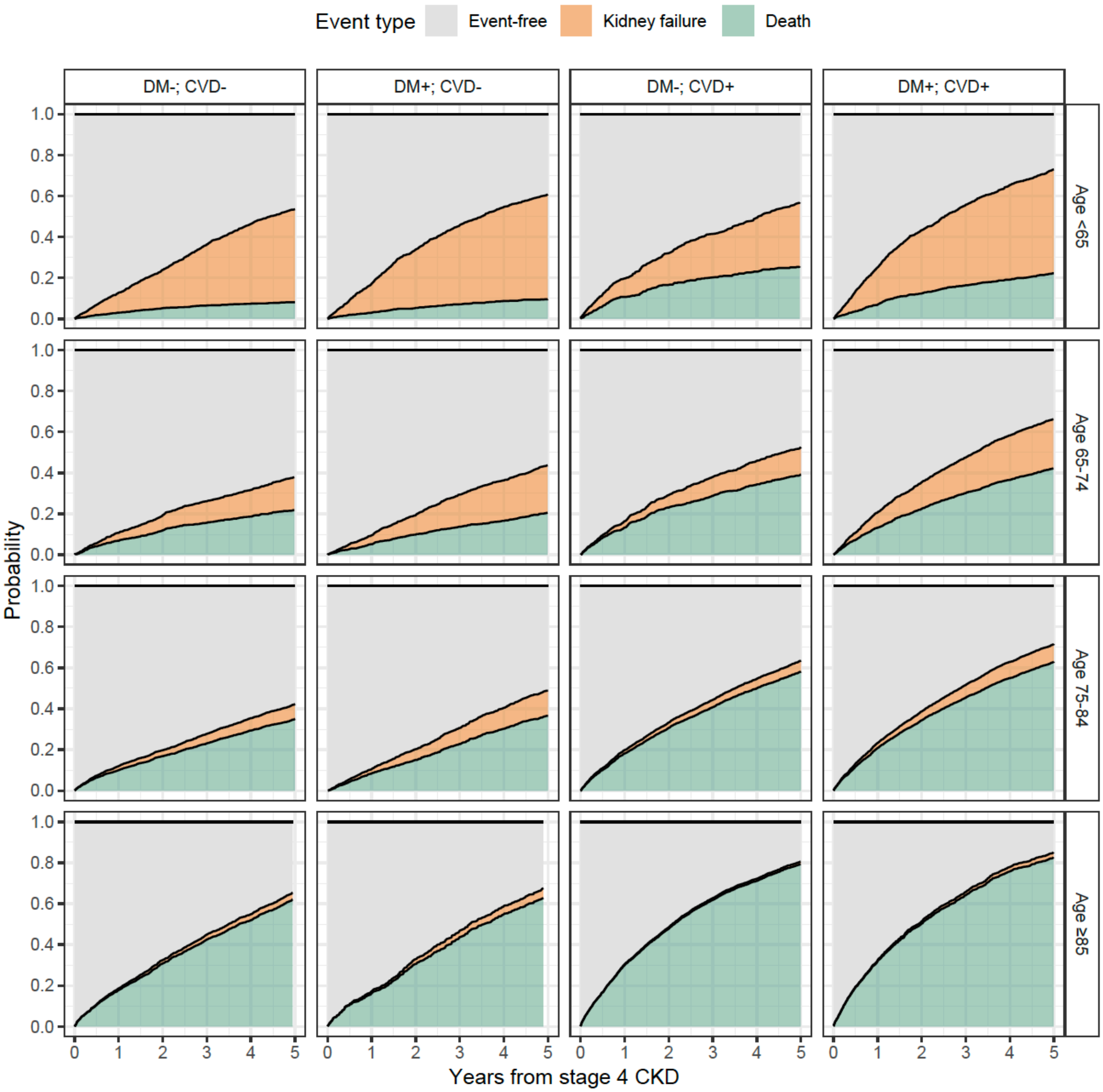

Legend: Stacked cumulative incidence functions (crude probabilities over time) stratified by age, diabetes (DM) and cardiovascular disease (CV). DM-/+ indicate absence (-) or presence of diabetes (DM); CV-/+ indicate absence (-) or presence of cardiovascular disease (CV). In this analysis kidney failure was defined as initiation of renal replacement therapy or sustained eGFR<10 ml/min/1.73 m^2 (occurrence of ≥2 consecutive eGFR values <10 ml/min/1.73m^2 over a period of >90 days).

eFigure 19. Stratified Probabilities (Sensitivity Analysis)

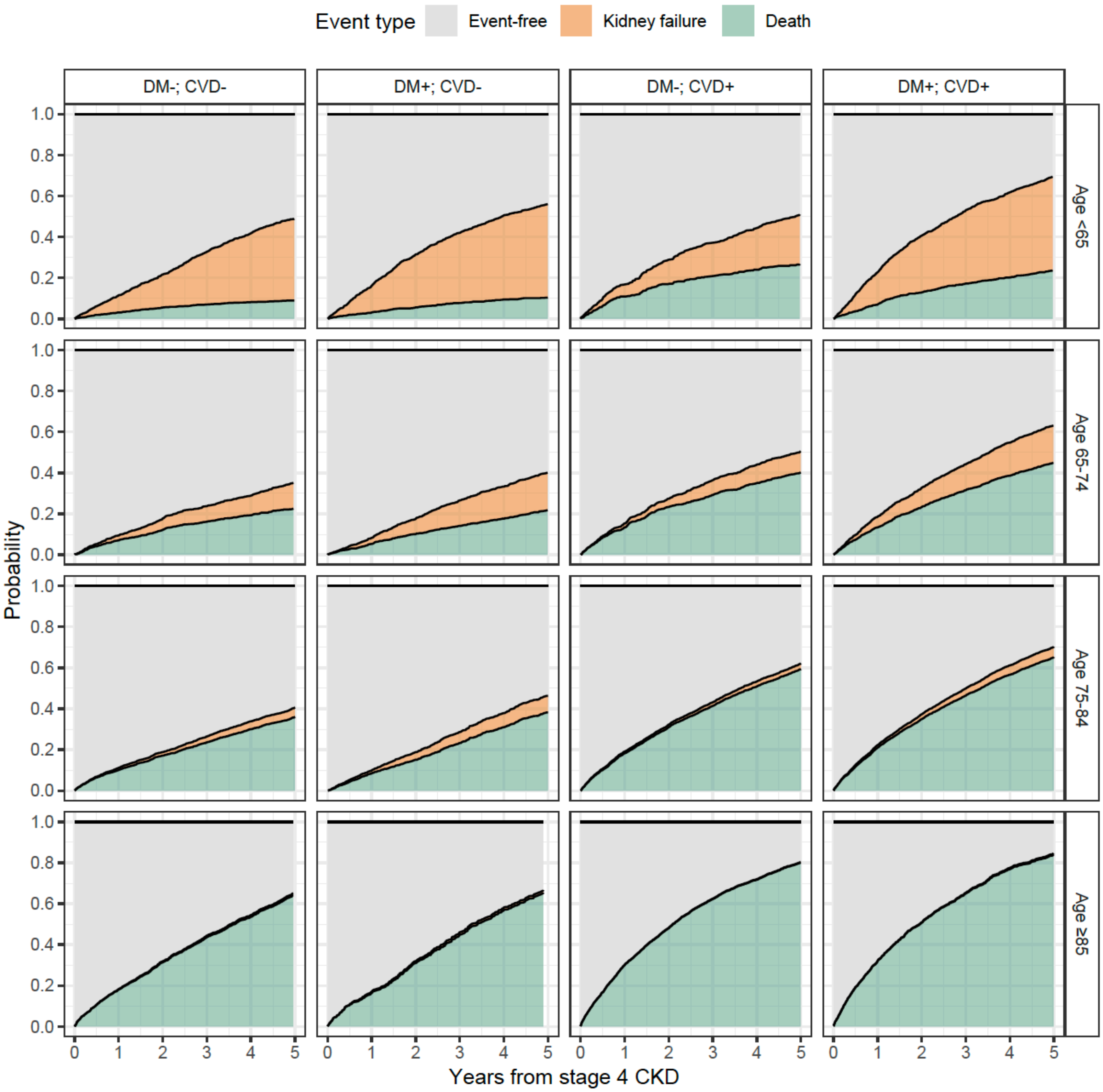

Legend: Stacked cumulative incidence functions (crude probabilities over time) stratified by age, diabetes (DM) and cardiovascular disease (CV). DM-/+ indicate absence (-) or presence of diabetes (DM); CV-/+ indicate absence (-) or presence of cardiovascular disease (CV). In this analysis kidney failure was defined as initiation of renal replacement therapy (receipt of a kidney transplant or registration in the provincial database of chronic dialysis).

©2020 Ravani P et al. *JAMA Network Open*

eFigure 20. Stratified Probabilities (Sensitivity Analysis)

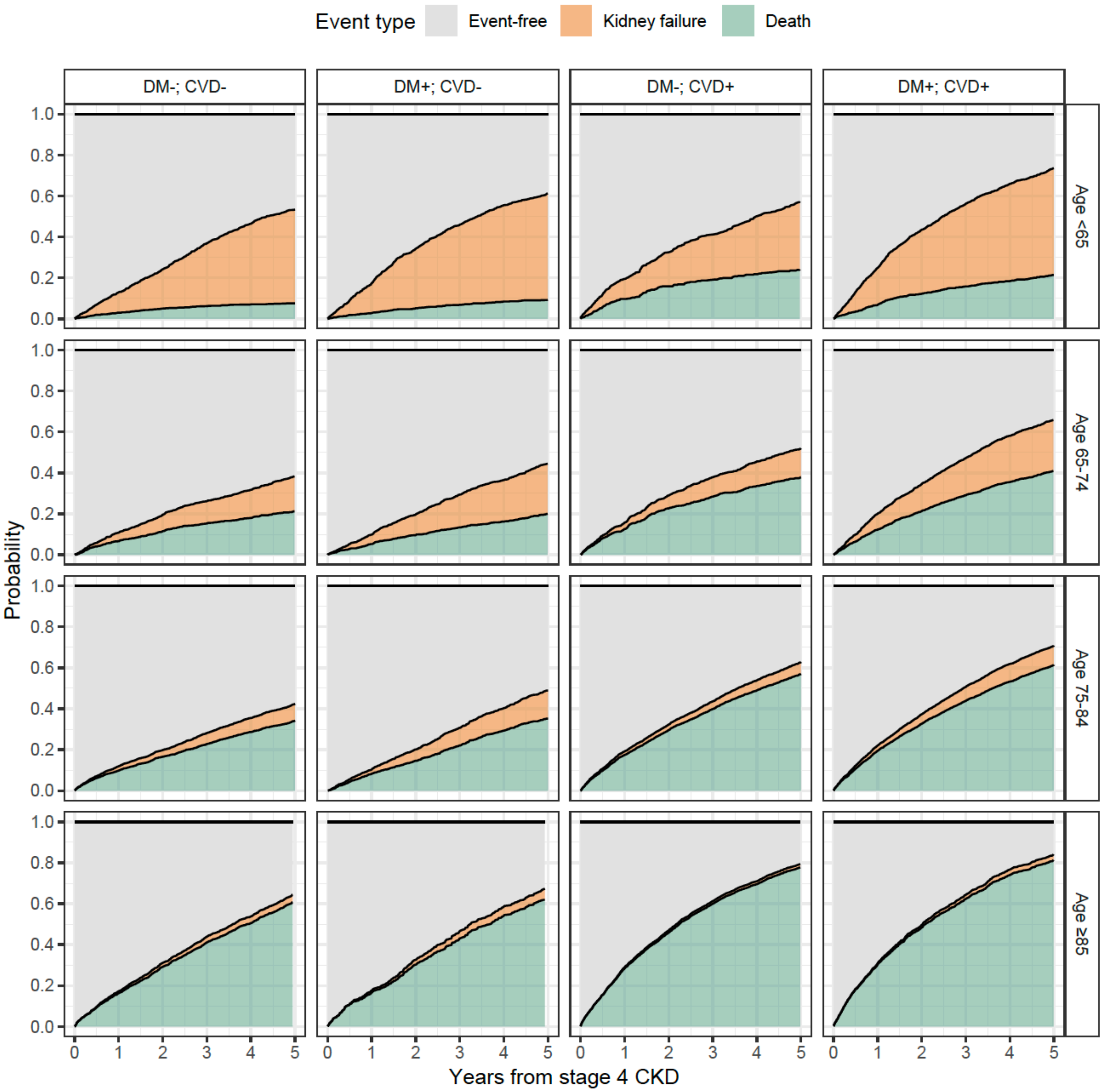

Legend: Stacked cumulative incidence functions (crude probabilities over time) stratified by age, diabetes (DM) and cardiovascular disease (CV). DM-/+ indicate absence (-) or presence of diabetes (DM); CV-/+ indicate absence (-) or presence of cardiovascular disease (CV). This analysis was restricted to complete cases for albuminuria.

eFigure 21. Clinical Scenarios 1

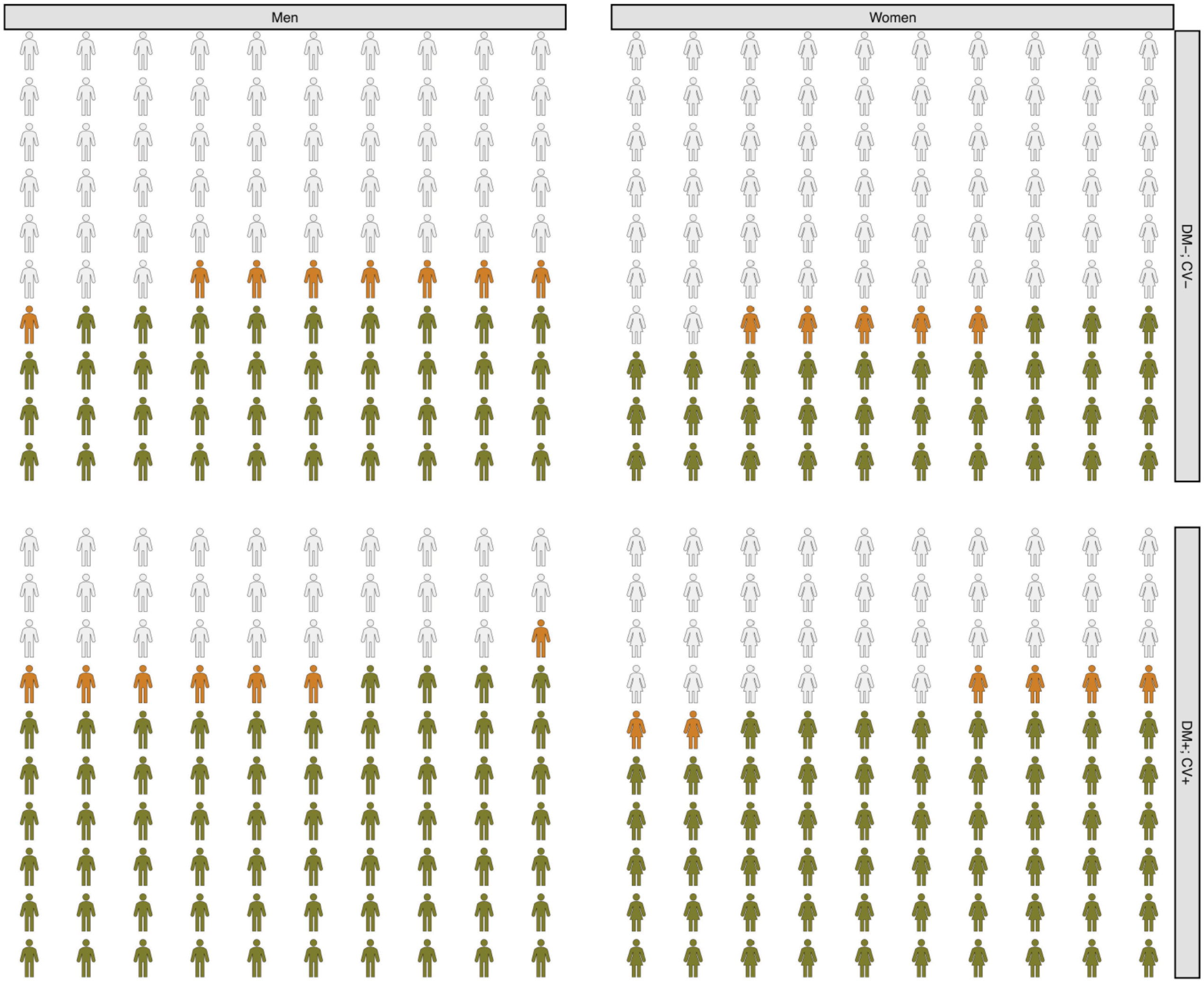

Expected 5-year model-based probabilities at age 80 years (median age of the study cohort) by sex and comorbidity, assuming absence of albuminuria (A1) and an eGFR of 25 ml/min/m^2. DM-/+ indicates absence (-) or presence of diabetes (DM); CV-/+ indicates absence (-) or presence of cardiovascular disease (CV). Light gray indicates ‘event-free’; orange indicates ‘kidney failure’; and green indicates ‘death’. At age 80y, the risk of kidney failure is small relative to the risk of death, regardless of sex or comorbid conditions commonly associated with chronic kidney disease. Additional clinical scenarios are presented in eFigure 21 in the Supplement.

©2020 Ravani P et al. *JAMA Network Open*

eFigure 22. Clinical Scenarios 2

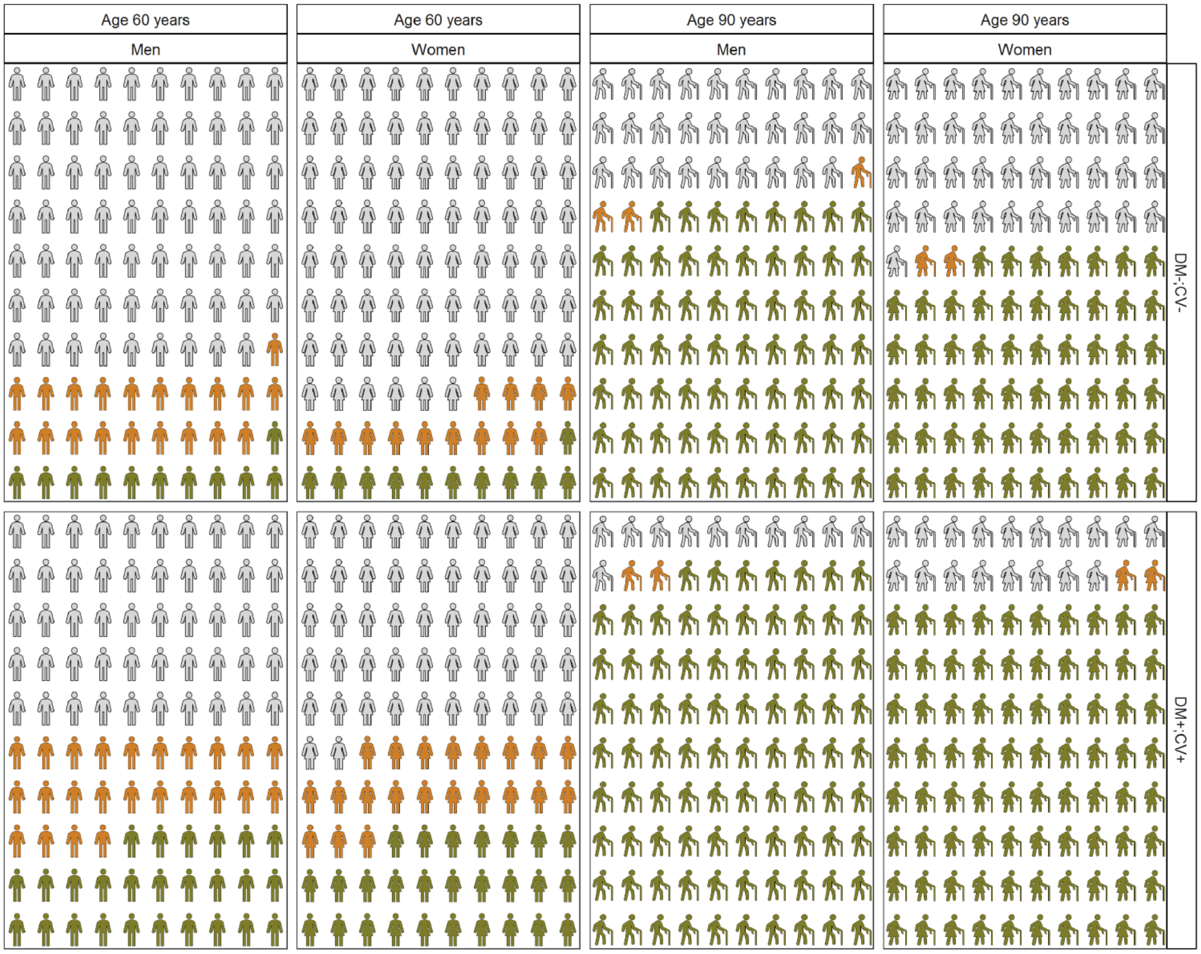

Legend: expected 5-year probabilities at age 60 and 90 years by comorbidity at baseline in men and women, assuming absence of albuminuria (A1) and an eGFR of 25 ml/min/m^2 (most likely values in the sample). DM-/+ indicates absence (-) or presence of diabetes (DM); CV-/+ indicates absence (-) or presence of cardiovascular disease (CV).

eReferences

1. Hemmelgarn BR, Clement F, Manns BJ, et al. Overview of the Alberta Kidney Disease Network. *BMC Nephrol.* 2009;10:30.
2. Hemmelgarn BR, James MT, Manns BJ, et al. Rates of treated and untreated kidney failure in older vs younger adults. *JAMA.* 2012;307(23):2507-2515.
3. Hemmelgarn BR, Manns BJ, Lloyd A, et al. Relation between kidney function, proteinuria, and adverse outcomes. *JAMA.* 2010;303(5):423-429.
4. Hemmelgarn BR, Zhang J, Manns BJ, et al. Progression of kidney dysfunction in the community-dwelling elderly. *Kidney Int.* 2006;69(12):2155-2161.
5. Liu P, Quinn RR, Karim ME, et al. Nephrology consultation and mortality in people with stage 4 chronic kidney disease: a population-based study. *CMAJ.* 2019;191(10):E274-E282.
6. Ishani A, Nelson D, Clothier B, et al. The magnitude of acute serum creatinine increase after cardiac surgery and the risk of chronic kidney disease, progression of kidney disease, and death. *Arch Intern Med.* 2011;171(3):226-233.
7. Moist LM, Fenton S, Kim JS, et al. Canadian Organ Replacement Register (CORR): reflecting the past and embracing the future. *Can J Kidney Health Dis.* 2014;1:26.
8. Tonelli M, Wiebe N, Fortin M, et al. Methods for identifying 30 chronic conditions: application to administrative data. *BMC Med Inform Decis Mak.* 2015;15:31.
9. KDIGO. Kidney Disease: Improving Global Outcomes (KDIGO CKD-Work-Group) 2012 Clinical Practice Guideline for the Evaluation and Management of Chronic Kidney Disease. *Kidney Int Suppl.* 2013;3(1):1-150.
10. Putter H, Fiocco M, Geskus RB. Tutorial in biostatistics: competing risks and multi-state models. *Statistics in Medicine.* 2007;26(11):2389-2430.
11. Prentice RL, Kalbfleisch JD, Peterson AV, Jr., Flournoy N, Farewell VT, Breslow NE. The analysis of failure times in the presence of competing risks. *Biometrics.* 1978;34(4):541-554.
12. Ozenne B SA, Scheike T, Torp-Pedersen C, Gerds TA. riskRegression: Predicting the Risk of an Event using Cox Regression Models. *The R Journal.* 2017;9(2):440-460.
13. de Wreede L, Fiocco M, Putter H. mstate: An R Package for the Analysis of Competing Risks and Multi-State Models. *Journal of Statistical Software.* 2011;7(38):1-30.
14. Gerds TA, Ozenne B. riskRegression: Risk Regression Models and Prediction Scores for Survival Analysis with Competing Risks. 2019.
15. Gray B. cmprsk: Subdistribution Analysis of Competing Risks. 2019.
